# Collection mode choice of spent electric vehicle batteries: considering collection competition and third-party economies of scale

**DOI:** 10.1038/s41598-022-10433-3

**Published:** 2022-04-23

**Authors:** Xin Li

**Affiliations:** School of Management Engineering, Shandong Jianzhu University, Jinan, 250101 China

**Keywords:** Environmental economics, Sustainability

## Abstract

With the rapid development of the electric vehicle (EV) industry, the recycling of spent EV batteries has attracted considerable attention. The establishment and optimization of the collection mode is a key link in regulating the recycling of spent EV batteries. This paper investigates an EV battery supply chain including an EV manufacturer, an EV retailer, and a third-party collector and analyzes three dual-channel collection modes. The optimal pricing and collection decisions of the three dual-channel collection modes are obtained and compared. The collection mode choice strategy and the effects of third-party economies of scale are explored. Three interesting insights are derived: (i) Third-party economies of scale can improve the collection rate of spent EV batteries and the profit of the supply chain. (ii) The optimal collection mode choice depends on the intensity of collection competition and the third-party economies of scale. (iii) When the intensity of collection competition and the third-party economies of scale are high enough, the EV retailer and the third-party dual-channel collection mode is the optimal mode; otherwise, the EV manufacturer and the EV retailer dual-channel collection mode is optimal.

## Introduction

Because environmental pollution and energy tensions are becoming increasingly tight, the new energy vehicle, especially the electric vehicle (EV), industry has developed rapidly worldwide because of its advantages in environmental protection and energy savings^1^. With the development of the industry, the derivative problem of EV battery recycling has become increasingly prominent^2–4^. It is estimated that retired EV batteries will reach 6 million packs by 2030^5^.

If such a large scale of retired EV batteries cannot be properly recycled, EV industrial development will encounter new bottlenecks^6^. Therefore, governments worldwide consider the management of EV battery recycling and improving the recycling policy system to be vital. In terms of lead-acid battery recycling, the United States, Japan, and the European Union have formulated recycling regulations based on the extended producer responsibility (EPR) system^7, 8^. China also follows the principle of the EPR system when formulating EV battery recycling policies^9^.

With the implementation of the EPR system, EV manufacturers employ different collection modes in practice^10, 11^. Some EV manufacturers collect the spent EV batteries through a single-channel mode. For example, Dongfeng Motor, one of the four major automobile groups in China, collects spent EV batteries through GEM (a third-party collector). By contrast, Jianghuai Motor provides incentives to retailers to induce the collection of spent EV batteries. Moreover, many EV manufacturers use dual-channel mode to collect spent EV batteries. For example, Soundon New Energy (an EV manufacturer) competes with his retailer on EV battery collection work. CATL (an EV battery manufacturer) competes with Hunan Brunp (a third-party collector) to perform collection activities. BYD 4S shop competes with GEM (a third-party collector) to promote the recycling of spent EV batteries^12^. BAIC (an EV manufacturer) competes with GEM to collect spent EV batteries.

The recycling of spent EV batteries has attracted considerable attention in the academic community. Many scholars focus on recycling technologies^13–15^, the second use of EV batteries^3, 16, 17^, the economic analysis of second use^18, 19^, energy and climate effects^20–22^, quick coding^23^, economic and environmental impacts^4, 24^. However, little research focuses on the collection mode choice strategy. Tang et al. analyzed three single-channel collection modes and three dual-channel collection modes under a reward-penalty mechanism^12^. They did not analyze the impacts of economies of scale. In practice, the difficulty of carrying out spent EV battery collection activities varies among the participants^25, 26^. Yi et al. and Han et al. assumed different collection costs to represent different difficulties of collection, where a lower collection cost represents lower collection difficulty^25, 26^. In the study of Lu et al., the lower collection cost is due to economies of scale^27^.

Based on the existing research, the competition among different collection channels and the third-party economies of scale are simultaneously considered in this paper. Three dual-channel collection modes are investigated: (i) the EV manufacturer competes with the EV retailer to collect spent EV batteries (M&R mode), (ii) the EV manufacturer competes with the third-party collector to collect spent EV batteries (M&TP mode), and (iii) the EV retailer competes with the third-party collector to collect spent EV batteries (R&TP mode). The aim of this paper is to answer the following questions: (i) How do third-party economies of scale influence the optimal decisions of different collection modes? (ii) How do competition intensity and third-party economies of scale affect the collection mode choice?

## Literature review

This study considers reverse channel management for spent EV battery collection. The related research is reviewed as follows.

### Literature on reverse channel management

Savaskan et al. compared three collection modes with a single reverse channel. Their research concluded that the retailer is the most effective collector of used products^28^. Hong and Yeh proposed a retailer collection model and a non-retailer (third-party collector) collection model. They found that retailer collection does not always outperform non-retailer collection. Retailer collection is dominant only if the third-party collector is a non-profit company^29^. Wang et al. considered the contract design problem for a manufacturer who entrusts collection to a retailer^30^. All the above studies focused on the single-channel collection mode.

Hong et al. and Hong and Yeh expanded the analysis to the dual-channel collection mode. They argued that the manufacturer and retailer dual-channel collection mode is the most effective mode for the manufacturer^29, 31^. Modak et al. analyzed the influences of recycling and product quality on pricing decisions in a closed loop supply chain (CLSC) with a three-channel collection mode: retailer-led collection, manufacturer-led collection, and third-party collector-led collection^32^. However, the above studies neglected the competition between different reverse channels.

### Literature on competition in reverse channels

Many studies have focused on reverse channel management with respect to competition between different channels. Competition between retailers has been investigated in some studies. Savaskan and Van Wassenhove explored the manufacturer’s channel choice considering the impacts of competition between two retailers^33^. De Giovanni took spent battery recycling as a case to analyze a joint maximization incentive in a CLSC with competing retailers^34^. Xu et al. investigated the situation in which two retailers compete on retailing products and recycling used products^35^.

Other studies investigate competition between a retailer and a third-party collector. Huang et al. analyzed the pricing decision and recycling strategies of a CLSC with dual collection channels, the retailer and third-party collector compete to collect the used products^36^, and compared their results with those of Savaskan et al.^28^. They found that when the competition intensity is within a certain range, the dual-channel collection mode outperforms the single-channel collection mode^28^. A CLSC with a similar structure was investigated in Wang et al. by considering asymmetric information and a reward-penalty mechanism^37^.

Other studies investigate competition among a manufacturer, retailer, and third-party collector. Based on Huang et al.^36^, Zhao et al. compared three single-channel collection modes (the manufacturer, the retailer and the third-party collector collection modes) with three dual-channel collection modes (the manufacturer competes with the retailer, the manufacturer competes with the third-party collector, and the retailer competes with the third-party collector)^38^. Liu et al. contended that the mode in which the manufacturer competes with the retailer is optimal for the manufacturer regardless of the competition intensity^39^. Liu and Zhang analyzed collection channel decisions under different power configurations^40^.

All the above-mentioned studies assumed that the collection difficulty of different collectors was the same. However, there are difference in the collection difficulty of different collectors.

### Literature on collection cost in reverse channels

The collection cost reflects the difference in the degree of difficulty in collection^27^. Some studies have explored the impacts of collection costs on manufacturers’ reverse channel choice^41–43^. Toyasaki et al.^44^ studied the impacts of recycling economies of scale on the choice of monopoly or competitive recycling. They found that when product substitution and economies of scale are weak, the competitive recycling model is favorable; when substitution is strong, recyclers tend to choose the monopolistic recycling model regardless of economies of scale^44^. A remanufacturer-dominated CLSC with multiple reverse channels was modeled in Huang et al., and the impacts of the economies of scale on reverse channel selection were analyzed^43^. Han et al. analyzed the manufacturer’s collection channel choice in a retailer-dominated CLSC with uncertain remanufacturing costs and found that direct recycling is more profitable than indirect recycling. Moreover, indirect recycling is more robust than the direct channel when considering remanufacturing risk^25^. Huang et al. found that reducing the reverse logistics cost coefficient and competition could improve profits in remanufacturing activities^45^.

Of the above studies, only Toyasaki et al. mentioned the competition between recyclers^44^. The competitive scheme implied that the recyclers compete indirectly through their contracted manufacturers instead of competing directly in the collection market. Studies on reverse channel choice considering different collection costs did not consider the competition between different collection channels. This paper attempts to fill this gap.

According to Lu et al., a lower collection cost comes from economies of scale^27^. Based on the studies of Liu et al. and Zhao et al., this paper further considers third-party economies of scale^38, 39^. Based on the studies of Han et al. and Yi et al., the completion between different collection channels is considered^25, 26^. The aim of this paper is to analyze the comprehensive impact of competition between different collection channels and third-party economies of scale on the optimal decisions of different collection modes and the collection mode choice strategy.

## Problem description

**Figure 1:**
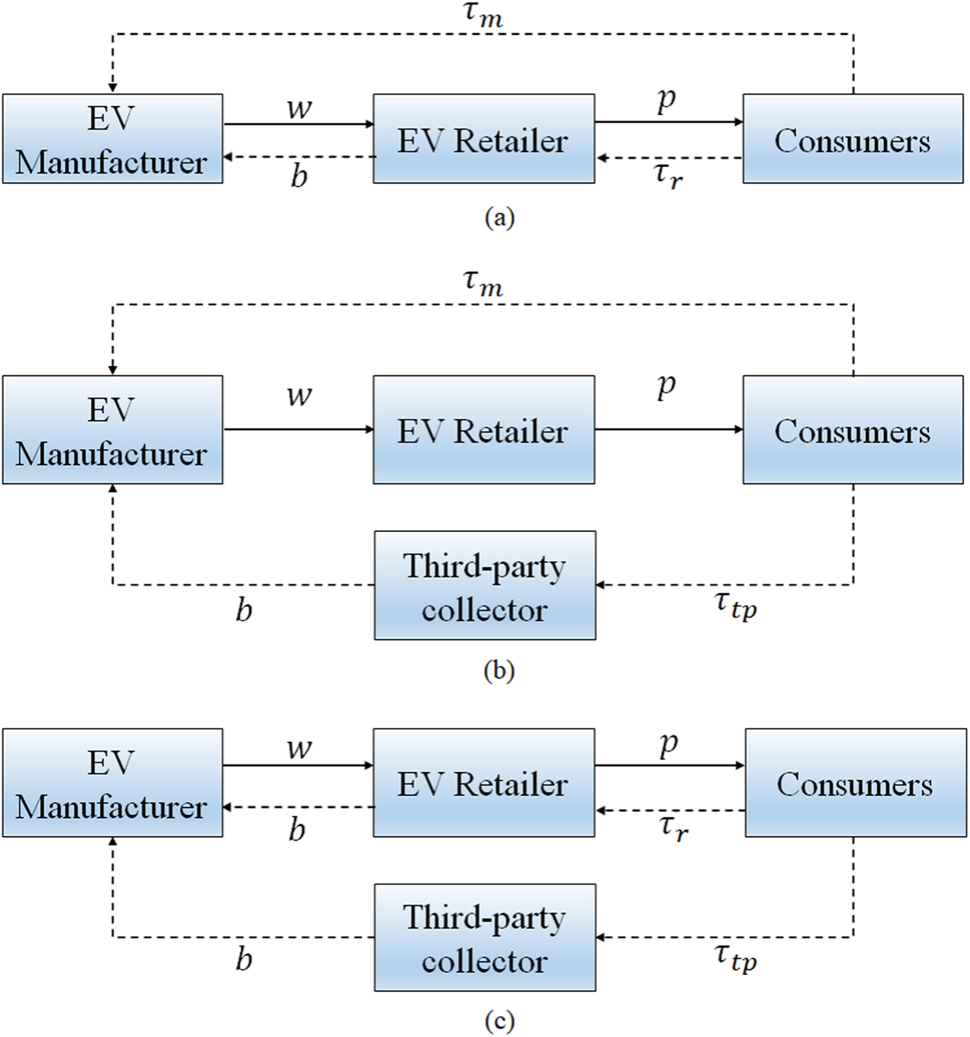
Three dual-channel collection modes: (**a**) the M&R mode; (**b**) the M&TP mode, and (**c**) the R&TP mode.

This paper investigates an EV supply chain composed of an EV manufacturer, an EV retailer, and a third-party collector. In the forward supply chain, the EV manufacturer sells EVs to the EV retailer. The EV retailer sells EVs to consumers. In the reverse supply chain, the EV manufacturer collects the spent EV batteries from consumers or from the EV retailer and the third-party collector. The EV manufacturer has three dual-channel collection mode options: the M&R mode, M&TP mode, and R&TP mode (Fig. 1).

### Notations

The notations are summarized in Table 1.

**Table 1:** Notations in the model.

Symbol	Definition
$\phi$	Potential demand
$\beta$	The sensitivity of consumers to the retail price
$p$	Unit retail price
$w$	Unit wholesale price
$c_{m}$	Unit cost of producing an EV battery with raw materials
$c_{r}$	Unit cost of producing an EV battery with recycled materials
$\Delta$	Unit benefit of remanufacturing
$b$	Unit transfer price of the manufacturer for returning a spent EV battery from the EV retailer/third-party collector
$C$	Scalar parameter
$\alpha$	Competition coefficient between two collection channels
$\delta$	The drop range of the collection investment of the third-party collector
$I_{i}$	The total collection investment of member $i$
$I_{i}^{*}$	The effective collection investment of member $i$
$\tau_{{_{i} }}^{j}$	The collection rate of member $i$ in mode $j$
$\pi_{{_{i} }}^{j}$	The profit of member $i$ in mode $j$

Subscript $i \in \{ M,R,TP,T\}$ represents the EV manufacturer, the EV retailer, the third-party collector, and the supply chain. Subscript $j \in \{ M\& R,M\& TP,R\& TP\}$ represents the M&R mode, the M&TP mode, and the R&TP mode.

### Assumptions

To establish the model, this paper makes the following assumptions:

i. All supply chain members are risk-neutral. The information is symmetrical among different members. All the members make decisions independently and attempt to maximize their own profit^11^.
ii. The EV manufacturer is the dominant leader and the other members are the followers in the EV supply chain^11^.
iii. The demand function is a linear function $D(p) = \phi - \beta p$, and it satisfies $D(c_{m} ) = \phi - \beta c_{m} > 0$, $\phi > \beta c_{m}$^28, 31, 39^.
iv. The remanufacturing process is profitable, namely $c_{r} < c_{m}$, and the unit savings in production cost by using recycled materials is $\Delta { = }c_{m} - c_{r}$. Therefore, the average production cost is $\overline{c} = (1 - \tau )c_{m} + \tau c_{r} = c_{m} - \tau \Delta$. In addition, the transfer price $b$ of the manufacturer for collecting batteries satisfies $0 \le b \le \Delta$^28, 31, 39^.
v. The collection investment of collector $i$ is $C\tau_{i}{^{2}}$^28^. Here, this paper assumes that the third-party collector has lower collection difficulty than the EV manufacturer and EV retailer because of its professional abilities. $C$ represents the scale parameter. Different $C$ values represent different investment costs under a given collection rate, which means that the larger $C$ is, the more difficult it is to collect^25, 26^.
vi. The parameter $\delta$($0 < \delta < 1$) is introduced to indicate the drop range of the third-party collector’s collection investment. Therefore, the collection investment function of the third-party collector is $\delta C\tau_{tp}{^{2}}$. The relatively lower collection investment is considered to be caused by the economies of scale of the third-party collector^27^.
vii. The collection rate of member $i$ can be expressed as $\tau_{i} = \sqrt {I_{i}^{*} /C}$, and the effective collection investment of member $i$ is $I_{i}^{*} = I_{i} - \alpha I_{j} , \;i \ne j$. To simplify the analysis, this paper assumes the same cross-influence effects for the collection investment among different collectors $\alpha_{M} = \alpha_{R} = \alpha_{TP} = \alpha , 0 \le \alpha \le 1$^33, 36, 39^.

This paper takes the M&TP mode as an example to illustrate the collection investment function:

1 $$I_{M} = \frac{{C(\tau_{m}{^{2}} + \alpha \delta \tau_{tp}{^{2}} )}}{{1 - \alpha^{2} }}, \; I_{tp} = \frac{{C(\delta \tau_{tp}{^{2}} + \alpha \tau_{m}{^{2}} )}}{{1 - \alpha^{2} }}$$

In addition, the total collection rate $0 \le \tau_{m} + \tau_{tp} \le 1$.

In the other two collection modes, the collection investment function of each member is consistent with Eq. (1).

## Model formulation and equilibriums in different modes

This section establishes game models and obtains the optimal decisions of the three dual-channel collection modes. The EV manufacturer behaves as the Stackelberg leader. The EV retailer and the third-party collector behave as followers and make the best responses to the EV manufacturer’s decisions.

### The M&R mode

In this mode, the EV manufacturer collects the spent EV batteries from consumers or from the EV retailer. First, the EV manufacturer determines its optimal wholesale price $w$, collection rate $\tau_{m}$, and transfer price $b$. Then, the retailer determines the retail price $p$ and the collection rate $\tau_{r}$.

In the M&R mode, the third-party collector is not involved in the system. Therefore, the optimal decisions of this mode have no relation to third-party economies of scale. The decision model and the optimal decisions are consistent with Liu et al.^39^ (see Table 2).

**Table 2:** The optimal decisions in the M&R mode.

Symbol	Value
$w^{{M{{\&}}R{*}}}$	$\frac{{[4C - \beta \Delta^{2} (1 - \alpha^{2} )(2 - \alpha {)}]\phi { + [}4C - \beta \Delta^{2} (1 - \alpha^{2} ){]}\beta c_{m} }}{{\beta [8C - \beta \Delta^{2} (1 - \alpha^{2} ){(3} - \alpha {)}]}}$
$b^{{M{{\&}}R*}}$	$\Delta$
$\tau_{m}^{{M{{\&}}R{*}}}$	$\frac{{\Delta (1 - \alpha^{2} )(\phi - \beta c_{{\text{m}}} )}}{{8C - \beta \Delta^{2} (1 - \alpha^{2} ){(3} - \alpha {)}}}$
$p^{{M{{\&}}R{*}}}$	$\frac{{[6C - \beta \Delta^{2} (1 - \alpha^{2} ){(3} - \alpha {)}]\phi { + }2C\beta c_{{\text{m}}} }}{{\beta [8C - \beta \Delta^{2} (1 - \alpha^{2} ){(3} - \alpha {)}]}}$
$\tau_{r}^{{M{{\&}}R{*}}}$	$\frac{{\Delta (1 - \alpha^{2} )(\phi - \beta c_{{\text{m}}} )}}{{8C - \beta \Delta^{2} (1 - \alpha^{2} ){(3} - \alpha {)}}}$
$\tau_{T}^{{M{{\&}}R*}}$	$\frac{{2\Delta (1 - \alpha^{2} )(\phi - \beta c_{{\text{m}}} )}}{{8C - \beta \Delta^{2} (1 - \alpha^{2} ){(3} - \alpha {)}}}$
$\pi_{m}^{{M{{\&}}R{*}}}$	$\frac{{C(\phi - \beta c_{{\text{m}}} )^{2} }}{{\beta [8C - \beta \Delta^{2} (1 - \alpha^{2} )(3 - \alpha {)}]}}$
$\pi_{r}^{{M{{\&}}R{*}}}$	$\frac{{C[4C - \beta \Delta^{2} (1 - \alpha^{2} )({1 + }\alpha {)}](\phi - \beta c_{{\text{m}}} )^{2} }}{{\beta [8C - \beta \Delta^{2} (1 - \alpha^{2} )(3 - \alpha {)}]^{2} }}$
$\pi_{T}^{{M{{\&}}R{*}}}$	$\frac{{4C[3C - \beta \Delta^{2} (1 - \alpha^{2} )](\phi - \beta c_{{\text{m}}} )^{2} }}{{\beta [8C - \beta \Delta^{2} (1 - \alpha^{2} )(3 - \alpha {)}]^{2} }}$

### The M&TP mode

In this mode, the EV manufacturer and the third-party collector take part in the collection work. First, the EV manufacturer makes the optimal decisions on the wholesale price $w$, collection rate $\tau_{m}$, and transfer price $b$. Then, the retailer determines the retail price $p$. Moreover, the third-party collector determines the collection rate $\tau_{tp}$.

The decision model of the M&TP mode is as follows:

2 $$\pi_{m}^{M\& TP} = [w - c_{m} + \Delta (\tau_{m} + \tau_{tp} )](\phi - \beta p) - \frac{{C\tau_{m}{^{2}} + \alpha \delta C\tau_{tp}{^{2}} }}{{1 - \alpha^{2} }} - b\tau_{tp} (\phi - \beta p)$$

3 $$\pi_{r}^{M\& TP} = (p - w)(\phi - \beta p)$$

4 $$\pi_{tp}^{M\& TP} = b\tau_{tp} (\phi - \beta p) - \frac{{\delta C\tau_{tp}{^{2}} + \alpha C\tau_{m}{^{2}} }}{{1 - \alpha^{2} }}$$

#### **Proposition 1**

*The optimal decisions of the EV manufacturer, the EV retailer, and the third-party collector in the M&TP mode can be obtained as follows*:

5 $$w^{{M{{\&}}TP*}} = \frac{{4C\delta (2 + \alpha )(\phi + \beta c_{m} ) - \beta \Delta^{2} \phi (1{ + }(2 + \alpha )\delta )(1 - \alpha^{2} )}}{{\beta [8C\delta (2 + \alpha ) - \beta \Delta^{2} (1{ + }(2 + \alpha )\delta )(1 - \alpha^{2} )]}}$$

6 $$\tau_{m}^{{M{{\&}}TP{*}}} = \frac{{\Delta \delta (2 + \alpha )(1 - \alpha^{2} )(\phi - \beta c_{m} )}}{{8C\delta (2 + \alpha ) - \beta \Delta^{2} (1 + (2 + \alpha )\delta )(1 - \alpha^{2} )}}$$

7 $$b^{{M{{\&}}TP*}} = \frac{\Delta }{2 + \alpha }$$

8 $$p^{{M{{\&}}TP{*}}} = \frac{{2C\delta (2 + \alpha )(3\phi + \beta c_{m} ) - \beta \Delta^{2} \phi (1{ + (2} + \alpha )\delta )(1 - \alpha^{2} )}}{{\beta [8C\delta (2 + \alpha ) - \beta \Delta^{2} (1 + {(2} + \alpha )\delta )(1 - \alpha^{2} )]}}$$

9 $$\tau_{tp}^{{M{{\&}}TP*}} = \frac{{\Delta (1 - \alpha^{2} )(\phi - \beta c_{m} )}}{{8C\delta (2 + \alpha ) - \beta \Delta^{2} (1 + (2 + \alpha )\delta )(1 - \alpha^{2} )}}$$

*From Eqs.* (5)–(9), *the total collection rate and the profits of the EV manufacturer, the EV retailer, the third-party collector, and the supply chain in the M&TP mode can be obtained as follows:*

10 $$\tau_{T}^{{M\& TP{*}}} = \frac{{\Delta (1{ + (}2 + \alpha )\delta )(1 - \alpha^{2} )(\phi - \beta c_{m} )}}{{8C\delta (2 + \alpha ) - \beta \Delta^{2} (1{ + (}2 + \alpha )\delta )(1 - \alpha^{2} )}}$$

11 $$\pi_{m}^{{M{{\&}}TP{*}}} = \frac{{C\delta (2 + \alpha )(\phi - \beta c_{m} )^{2} }}{{\beta [8C\delta (2 + \alpha ) - \beta \Delta^{2} (1 + (2 + \alpha )\delta )(1 - \alpha^{2} )]}}$$

12 $$\pi_{r}^{{M{{\&}}TP{*}}} = \frac{{4C^{2} \delta^{2} (2 + \alpha )^{2} (\phi - \beta c_{m} )^{2} }}{{\beta [8C\delta (2 + \alpha ) - \beta \Delta^{2} (1{ + (}2 + \alpha )\delta )(1 - \alpha^{2} )]^{2} }}$$

13 $$\pi_{tp}^{{M{{\&}}TP{*}}} = \frac{{C\delta \Delta^{2} [1 - \alpha \delta (2 + \alpha )^{2} ](\phi - \beta c_{m} )^{2} (1 - \alpha^{2} )}}{{[8C\delta (2 + \alpha ) - \beta \Delta^{2} (1{ + }(2 + \alpha )\delta )(1 - \alpha^{2} )]^{2} }}$$

14 $$\pi_{T}^{{M{{\&}}TP{*}}} = \frac{{(12C^{2} \delta^{2} (2 + \alpha )^{2} - \beta \Delta^{2} C\delta (1 + \alpha )(1 + (2 + \alpha )^{2} \delta )(1 - \alpha^{2} ))(\phi - \beta c_{m} )^{2} }}{{\beta [8C\delta (2 + \alpha ) - \beta \Delta^{2} (1 + (2 + \alpha )\delta )(1 - \alpha^{2} )]^{2} }}$$

### The R&TP mode

In this collection mode, the EV manufacturer does not directly collect spent EV batteries from consumers. Instead, it retrieves them through the EV retailer and the third-party collector. First, the EV manufacturer determines the wholesale price $w$, and the transfer price $b$. Then, the EV retailer determines the retail price $p$ and collection rate $\tau_{r}$. Moreover, the third-party collector determines the collection rate $\tau_{tp}$.

The decision model of the R&TP mode is given as follows:

15 $$\pi_{m}^{R\& TP} = [w - c_{m} + \Delta (\tau_{r} + \tau_{tp} )](\phi - \beta p) - b\tau_{r} (\phi - \beta p) - b\tau_{tp} (\phi - \beta p)$$

16 $$\pi_{r}^{R\& TP} = (p - w)(\phi - \beta p) + b\tau_{r} (\phi - \beta p) - \frac{{C\tau_{r}^{2} + \alpha \delta C\tau_{tp}^{2} }}{{1 - \alpha^{2} }}$$

17 $$\pi_{tp}^{R\& TP} = b\tau_{tp} (\phi - \beta p) - \frac{{\delta C\tau_{tp}^{2} + \alpha C\tau_{r}^{2} }}{{1 - \alpha^{2} }}$$

#### **Proposition 2**

*The optimal decisions of the EV manufacturer, the EV retailer, and the third-party collector in the R&TP mode can be obtained as follows:*

18 $$w^{{R{{\&}}TP*}} = \frac{\phi }{\beta } - \frac{{\delta (16C - (1 - \alpha^{2} )\beta \Delta^{2} (1{ + }\delta )^{2} )(\phi - \beta c_{m} )}}{{2\beta [16C\delta - (1 - \alpha^{2} )\beta \Delta^{2} (1{ + }\delta )^{2} ]}}$$

19 $$b^{{R{{\&}}TP*}} = \frac{(1 + \delta )\Delta }{2}$$

20 $$p^{{R{{\&}}TP{*}}} = \frac{{(12C\delta - (1 - \alpha^{2} )\beta (1 + \delta )^{2} \Delta^{2} )\phi + 4C\beta \delta c_{m} }}{{\beta (16C\delta - (1 - \alpha^{2} )\beta (1 + \delta )^{2} \Delta^{2} )}}$$

21 $$\tau_{r}^{{R{{\&}}TP{*}}} = \frac{{(1 - \alpha^{2} )\delta (1 + \delta )\Delta (\phi - \beta c_{m} )}}{{16C\delta - (1 - \alpha^{2} )\beta (1 + \delta )^{2} \Delta^{2} }}$$

22 $$\tau_{tp}^{{R{{\&}}TP*}} = \frac{{(1 - \alpha^{2} )(1 + \delta )\Delta (\phi - \beta c_{m} )}}{{16C\delta - (1 - \alpha^{2} )\beta (1 + \delta )^{2} \Delta^{2} }}$$

*From* Eqs. (18)–(22), *the total collection rate and the profits of the EV manufacturer, the EV retailer, the third-party collector, and the supply chain in the R&TP mode can be obtained as follows:*

23 $$\tau_{T}^{{R\& TP{*}}} = \frac{{(1 - \alpha^{2} )(1 + \delta )^{2} \Delta (\phi - \beta c_{m} )}}{{16C\delta - (1 - \alpha^{2} )\beta (1 + \delta )^{2} \Delta^{2} }}$$

24 $$\pi_{m}^{{R{{\&}}TP*}} = \frac{{2C\delta (\phi - \beta c_{m} )^{2} }}{{\beta [16C\delta - \beta \Delta^{2} (1 + \delta )^{2} (1 - \alpha^{2} )]}}$$

25 $$\pi_{r}^{{R{{\&}}TP{*}}} = \frac{{C\delta (16C\delta - (1 - \alpha^{2} )\beta (1 + \delta )^{2} (\delta + \alpha )^{2} \Delta^{2} )(\phi - \beta c_{m} )^{2} }}{{\beta [16C\delta - \beta \Delta^{2} (1 + \delta )^{2} (1 - \alpha^{2} )]^{2} }}$$

26 $$\pi_{tp}^{{R{{\&}}TP{*}}} = \frac{{C\delta \Delta^{2} (1 - \alpha^{2} )(1 + \delta )^{2} (1 - \alpha \delta )(\phi - \beta c_{m} )^{2} }}{{[16C\delta - \beta \Delta^{2} (1 + \delta )^{2} (1 - \alpha^{2} )]^{2} }}$$

27 $$\pi_{T}^{{R{{\&}}TP{*}}} = \frac{{C\delta (48C\delta - (1 - \alpha^{2} )(1 + \alpha )\beta (1 + \delta )^{3} \Delta^{2} )(\phi - \beta c_{m} )^{2} }}{{\beta [16C\delta - \beta \Delta^{2} (1 + \delta )^{2} (1 - \alpha^{2} )]^{2} }}$$

## Results and discussion

The following analysis is based on the data in existing references^36, 38, 39^. The parameters are $C = 1000$, $\phi { = }100$, $\beta { = }0.3$, $c_{m} = 20$, and $\Delta { = }15$.

### Comparison of different modes

In this section, the optimal decisions of the three dual-channel collection modes are compared, and the reverse channel choice is explored from the perspectives of the collection rate and the total profit. In addition, the effects of third-party economies of scale are investigated.

#### The retail price

##### **Corollary 1**

*The retail prices in the three dual-channel collection modes are ordered as follows:*

$$\left\{ \begin{aligned} & p^{M\& R*} > p^{M\& TP*} > p^{R\& TP*} \quad if \;\; 0 < \delta < \frac{{1 - \alpha^{2} }}{{4 - \alpha^{2} (5 - \alpha^{2} )}} \hfill \\ & p^{M\& TP*} > p^{M\& R*} > p^{R\& TP*} \quad if \;\; \frac{{1 - \alpha^{2} }}{{4 - \alpha^{2} (5 - \alpha^{2} )}} < \delta < 2 - \alpha - \sqrt {3 - 4\alpha + \alpha^{2} } \hfill \\ & p^{M\& TP*} > p^{R\& TP*} > p^{M\& R*} \quad if \;\; 2 - \alpha - \sqrt {3 - 4\alpha + \alpha^{2} } < \delta < 1 \hfill \\ \end{aligned} \right.$$

##### *Proof*

See supplementary materials, Proof of Corollary 1.

**Figure 2:**
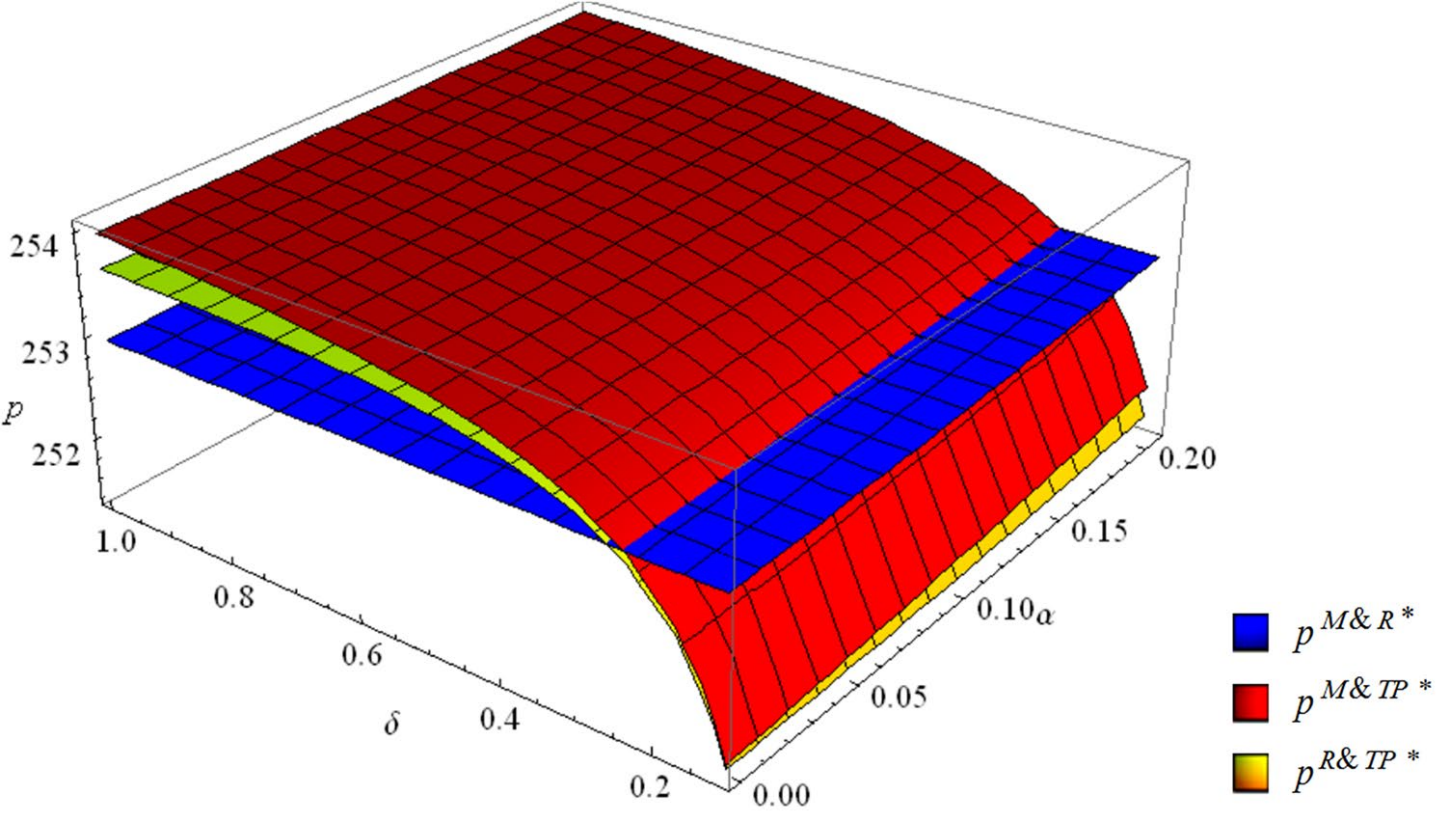
The retail prices in the three collection modes.

Figure 2 indicates that the retail price in the R&TP mode is the lowest when $\delta < 2 - \alpha - \sqrt {3 - 4\alpha + \alpha^{2} }$. When $2 - \alpha - \sqrt {3 - 4\alpha + \alpha^{2} } < \delta$, the retail price in the M&R mode is the lowest among the three collection modes. When ${{(1 - \alpha^{2} )} \mathord{\left/ {\vphantom {{(1 - \alpha^{2} )} {[4 - \alpha^{2} (5 - \alpha^{2} )]}}} \right. \kern-\nulldelimiterspace} {[4 - \alpha^{2} (5 - \alpha^{2} )]}} < \delta$, the retail price in the M&TP mode is the highest. When $\delta <{{(1 - \alpha^{2} )} \mathord{\left/ {\vphantom {{(1 - \alpha^{2} )} {[4 - \alpha^{2} (5 - \alpha^{2} )]}}} \right. \kern-\nulldelimiterspace} {[4 - \alpha^{2} (5 - \alpha^{2} )]}}$, the M&R mode has the highest retail price.

#### The collection transfer price

The unit transfer price reflects the unit collection price paid by the EV manufacturer to the EV retailer and the third-party collector to retrieve spent EV batteries. The optimal transfer prices of the EV manufacturer in the three collection modes are ordered as follows: $b^{{M{{\&}}R*}} > b^{{R{{\&}}TP*}} > b^{{M{{\&}}TP*}}$.

##### *Proof*

$$\left\{ \begin{aligned} & b^{M\& R*} - b^{R\& TP*} = \frac{(1 - \delta )\Delta }{2} > 0 \hfill \\ & b^{R\& TP*} - b^{M\& TP*} = \frac{\Delta }{2}\left( {\frac{\alpha }{2 + \alpha } + \delta } \right) > 0 \hfill \\ \end{aligned} \right.$$

The findings indicate that regardless of what the intensity of competition and the third-party economies of scale are, the transfer price of the EV manufacturer in the M&R mode is the highest among the three collection modes. The competition between dual channels and the third-party economies of scale has no effect on the ranking of the EV manufacturer’s transfer prices^39^.

#### The EV manufacturer’s profit

##### **Corollary 2**

*The EV manufacturer dominates the supply chain and is profit-oriented. The EV manufacturer’s profits in the three modes are ordered as follows:*

$$\left\{ \begin{aligned} & \pi_{m}^{M\& TP*} < \pi_{m}^{R\& TP*} < \pi_{m}^{M\& R*} \quad if \; \; 2 - \alpha - \sqrt {(3 - \alpha )(1 - \alpha )} < \delta < 1 \hfill \\ & \pi_{m}^{M\& TP*} < \pi_{m}^{M\& R*} < \pi_{m}^{R\& TP*} \quad if \;\; \frac{1}{{4 - \alpha^{2} }} < \delta < 2 - \alpha - \sqrt {(3 - \alpha )(1 - \alpha )} \hfill \\ & \pi_{m}^{M\& R*} < \pi_{m}^{M\& TP*} < \pi_{m}^{R\& TP*} \quad if \;\; 0 < \delta < \frac{1}{{4 - \alpha^{2} }} \hfill \\ \end{aligned} \right.$$

##### *Proof*

See supplementary materials, Proof of Corollary 2.

**Figure 3:**
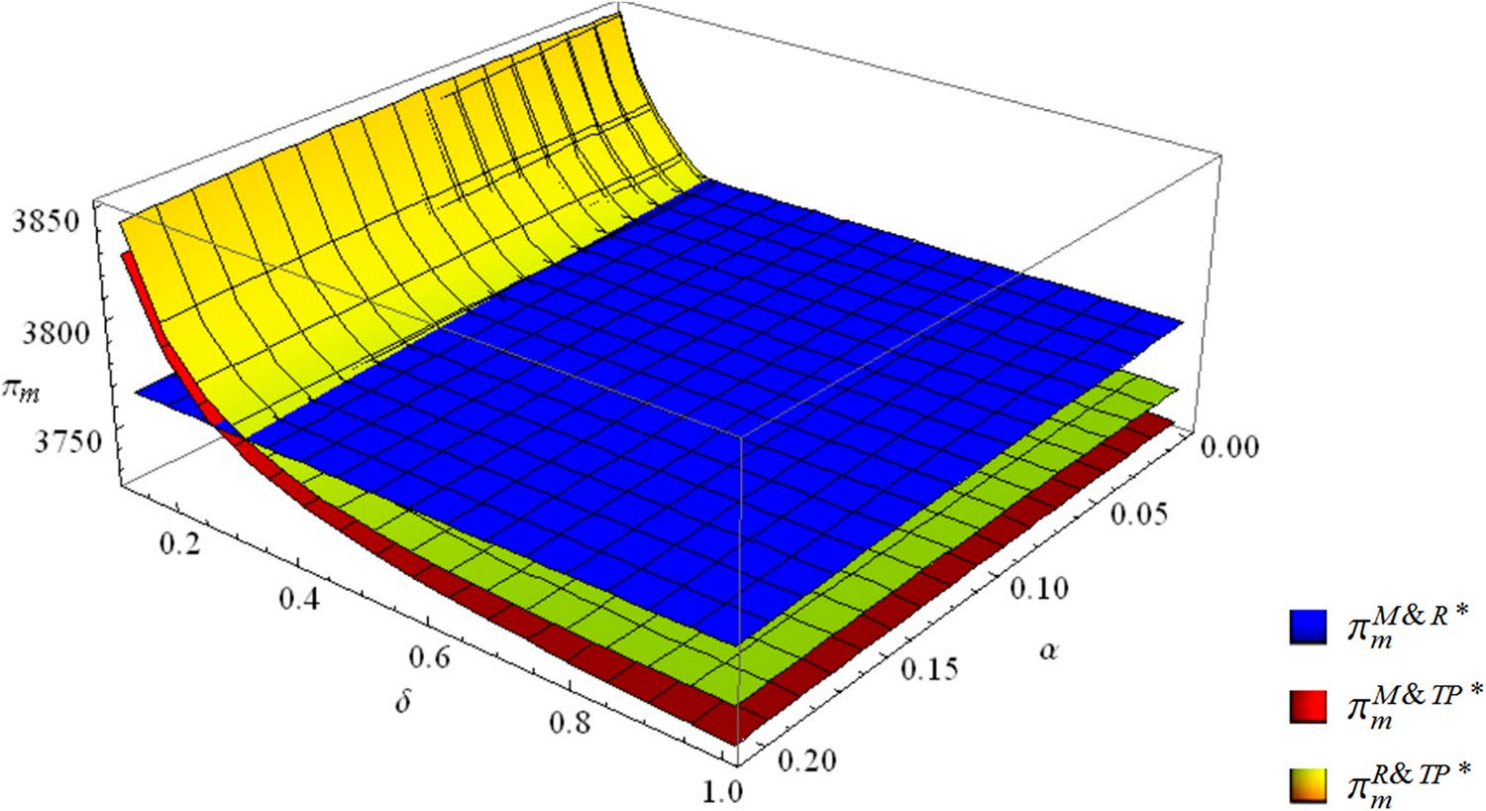
The profits of the EV manufacturer in the three collection modes.

Figure 3 shows that the ranking of the EV manufacturer’s profits in the three dual-channel collection modes varies with the competition intensity and the third-party economies of scale. The EV manufacturer’s profit in the M&R mode is highest when $\delta > 2 - \alpha - \sqrt {(3 - \alpha )(1 - \alpha )}$, which means that the EV manufacturer and the EV retailer engaging in collection activities is suitable for the EV manufacturer in this scenario. Otherwise, when $\delta < 2 - \alpha - \sqrt {(3 - \alpha )(1 - \alpha )}$, the R&TP mode has the greatest profit. Therefore, in this scenario, the EV manufacturer tends to outsource collection to the EV retailer and the third-party collector.

### The collection mode choice

#### From the perspective of the total profit

**Figure 4:**
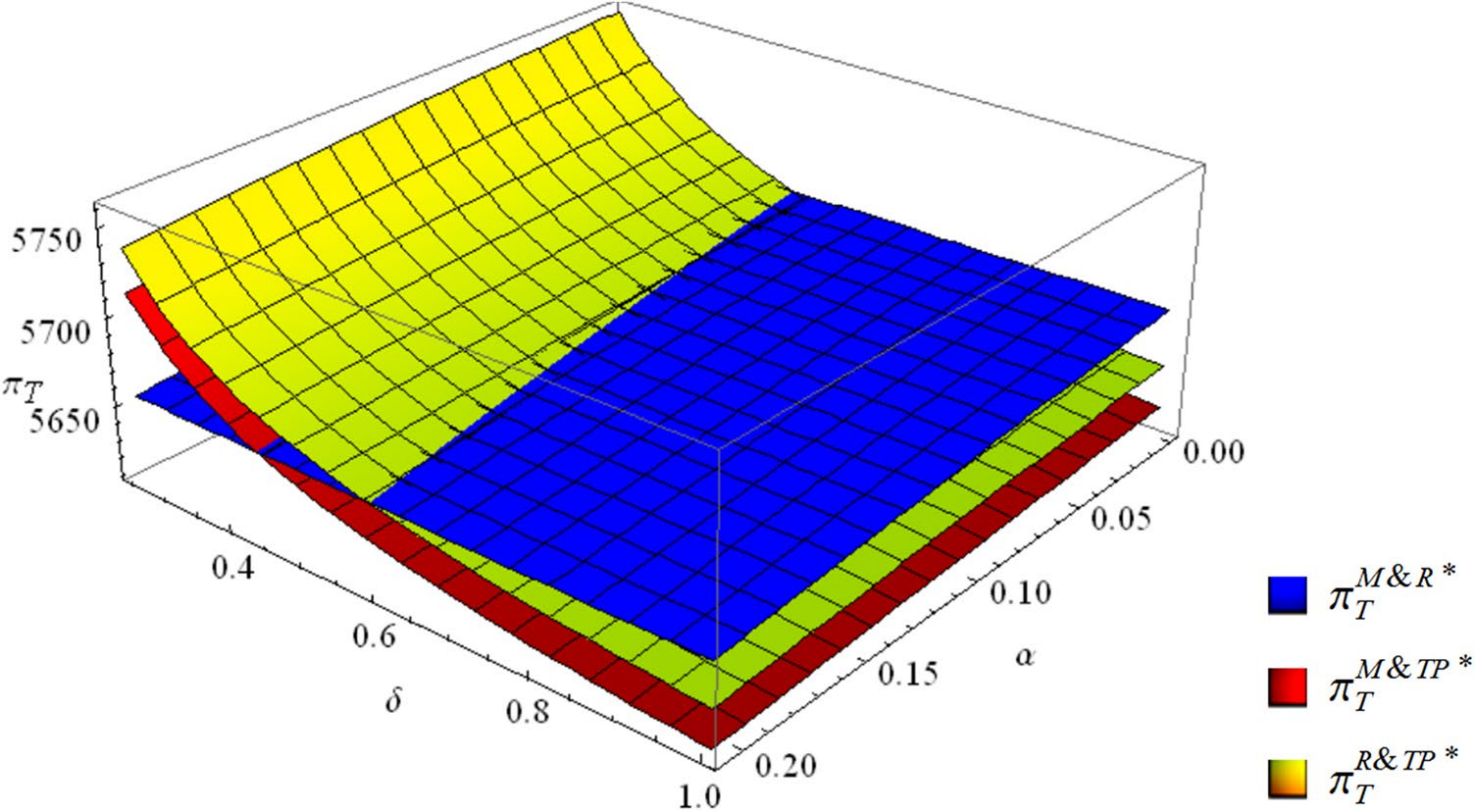
The total profits of the three collection modes.

The profit of the supply chain is related as follows: when the competition for collection is weak and the third-party economies of scale are low, the M&R mode has the highest profit. Otherwise, the R&TP mode has the highest profit. Since the profit function is a higher-order function with respect to $\alpha$ and $\delta$, the formula for the critical line is too complicated. This paper uses Fig. 4 to show the above conclusion visually.

For the whole supply chain, when collection competition is intensified, the R&TP mode is beneficial only when the third-party economies of scale are high enough to compensate for the increase in the collection cost caused by competition. Otherwise, the M&R mode is more profitable.

Therefore, from the perspective of total profit, the R&TP mode is the optimal collection mode when the third-party economies of scale are high. The M&R mode is the optimal collection mode when the third-party economies of scale are low.

#### From the perspective of the total collection rate

##### **Corollary 3**

*The environmental impact is reflected by the total collection rate. The total collection rates in the three modes are related as follows:*

$$\left\{ \begin{aligned} & \tau_{T}^{M\& R*} > \tau_{T}^{R\& TP*} > \tau_{T}^{M\& TP*} \quad if\;\; \frac{{120 - \alpha - \alpha^{2} - 22\sqrt {1 - \alpha - \alpha^{2} } }}{{118 + \alpha + \alpha^{2} }} < \delta < 1 \hfill \\ & \tau_{T}^{R\& TP*} > \tau_{T}^{M\& R*} > \tau_{T}^{M\& TP*} \quad if \;\; \frac{{118 + \alpha + \alpha^{2} }}{{240 + 118\alpha - 3\alpha^{2} }} < \delta < \frac{{120 - \alpha - \alpha^{2} - 9\sqrt {1 - \alpha - \alpha^{2} } }}{{118 + \alpha + \alpha^{2} }} \hfill \\ & \tau_{T}^{R\& TP*} > \tau_{T}^{M\& TP*} > \tau_{T}^{M\& R*} \quad if \;\; 0 < \delta < \frac{{118 + \alpha + \alpha^{2} }}{{240 + 118\alpha - 3\alpha^{2} }} \hfill \\ \end{aligned} \right.$$

##### *Proof*

See supplementary materials, Proof of Corollary 3.

**Figure 5:**
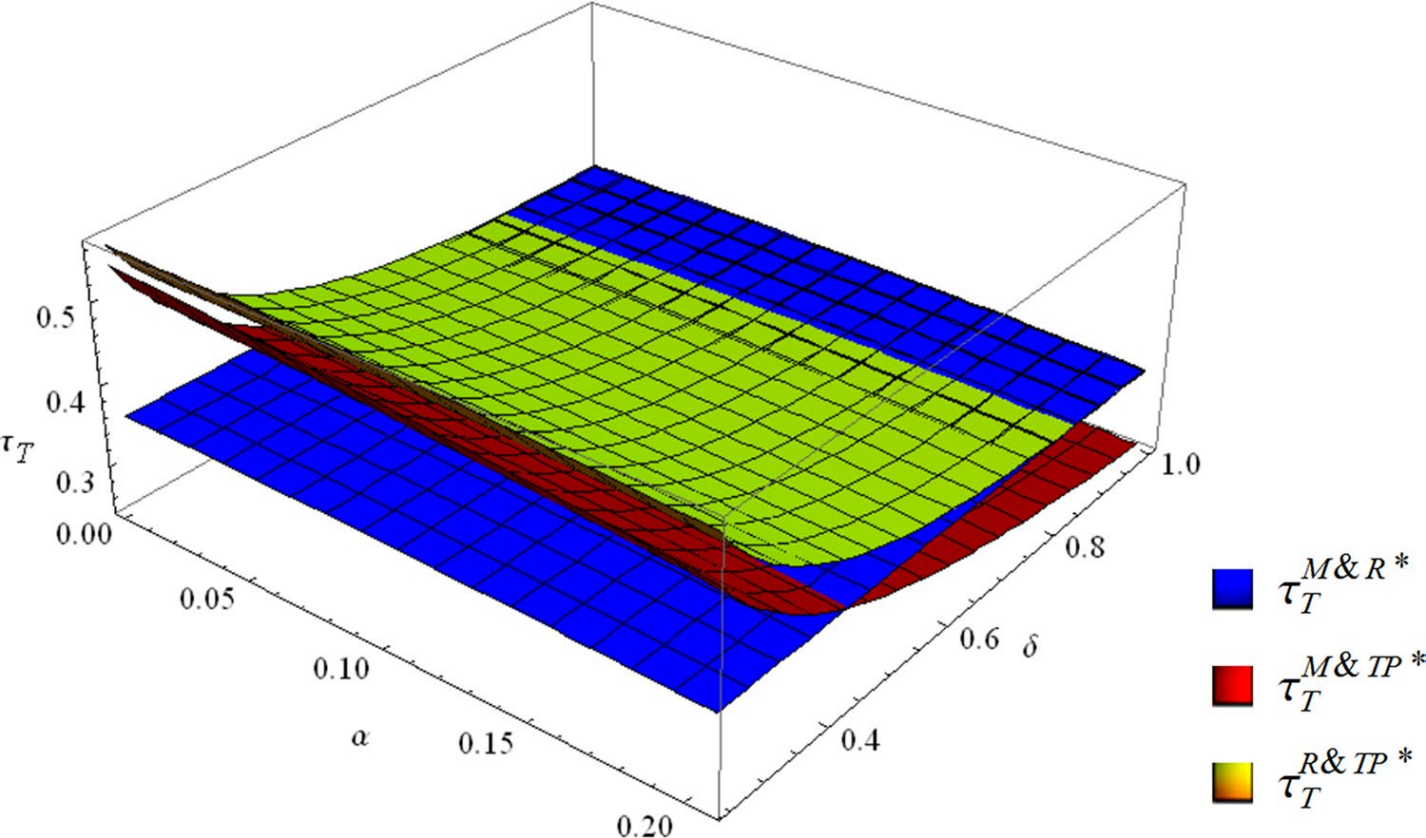
The total collection rates of the three collection modes.

Figure 5 vividly illustrates the above conclusion.

Figure 5 shows that the total collection rate in the M&R mode is gradually reduced as the competition between different collection channels intensifies. The total collection rates in the M&TP and R&TP modes increase efficiently and gradually outperform that of the M&R mode with the improvement in third-party economies of scale. In addition, the collection rate in the R&TP mode is always higher than that in the M&TP mode. When $\delta < {{(118 + \alpha + \alpha^{2} )} \mathord{\left/ {\vphantom {{(118 + \alpha + \alpha^{2} )} {(240 + 118\alpha - 3\alpha^{2} )}}} \right. \kern-\nulldelimiterspace} {(240 + 118\alpha - 3\alpha^{2} )}}$, the R&TP mode has the highest total collection rate and is superior to the other two modes; otherwise, the M&R mode is dominant.

In general, from the perspective of the total collection rate, when the third-party economies of scale are high, the R&TP mode is the optimal collection mode. When the third-party economies of scale are low, the M&R mode is the optimal collection mode.

### Effect of the third-party economies of scale

To explore the impact of the third-party economies of scale, the decisions in the M&TP mode and the R&TP mode are compared with the results in existing research^39^ that disregarded third-party economies of scale (see Table S1). The values with subscripts M&3P* and R&3P* represent the corresponding results in Liu et al.^39^.

#### Impact on the M&TP mode

##### The price decisions

###### **Corollary 4**

*The wholesale price, retail price, and transfer price in the two cases (with or without third-party economies of scale) are related as follows:* $w^{M\& 3P*} > w^{M\& TP*}$, $p^{M\& 3P*} > p^{M\& TP*}$, $b^{M\& 3P*} = b^{M\& TP*}$.

###### *Proof*

See supplementary materials, Proof of Corollary 4.

This indicates that the wholesale price and retail price decrease under the impact of third-party economies of scale. However, the transfer price remains unchanged.

The third-party economies of scale increase the total collection rate, resulting in a decline in the average production cost, which in turn causes the EV manufacturer and the EV retailer to lower the wholesale price and the retail price. However, the third-party economies of scale do not affect the transfer price of the EV manufacturer.

##### The collection rate decisions

###### **Corollary 5**

*The collection rates of the EV manufacturer, the third-party collector, and the supply chain are related as follows:* $\tau_{m}^{M\& 3P*} > \tau_{m}^{M\& TP*}$,$\tau_{tp}^{M\& 3P*} < \tau_{tp}^{M\& TP*}$ and $\tau_{T}^{M\& 3P*} < \tau_{T}^{M\& TP*}$.

###### *Proof*

See supplementary materials, Proof of Corollary 5.

This indicates that the total collection rate can be improved by third-party economies of scale. Specifically, economies of scale increase the collection rate of the third-party collector, and lower the collection rate of the EV manufacturer. Overall, the increase in the third-party collector’s collection rate can compensate for the decrease in the EV manufacturer’s collection rate.

The economies of scale result in a lower average collection cost of the third-party collector, leading to an advantage in the game with the EV manufacturer and resulting in a lower collection rate of the EV manufacturer. Moreover, the marginal cost of increasing the collection rate for the third-party collector is much lower than that of the EV manufacturer. Therefore, the total collection rate of the supply chain is still improved even when the collection rate of the EV manufacturer decreases.

##### The profits of each member and the supply chain

###### **Corollary 6**

*The profits of the EV manufacturer, the EV retailer, the third-party collector, and the supply chain are ordered as follows:* $\pi_{m}^{M\& 3P*} < \pi_{m}^{M\& TP*}$, $\pi_{r}^{M\& 3P*} < \pi_{r}^{M\& TP*}$, $\pi_{3p}^{M\& 3P*} < \pi_{TP}^{M\& TP*}$, $\pi_{T}^{M\& 3P*} < \pi_{T}^{M\& TP*}$.

###### *Proof*

See supplementary materials, Proof of Corollary 6.

Thus, third-party economies of scale can improve the profit of each member and the supply chain.

Third-party economies of scale raise the total collection rate, which reduces the average production cost. The EV manufacturer transfers the marginal profit in the reproduction process to the EV retailer by reducing the wholesale price. Furthermore, demand rises as the EV retailer decreases the retail price, resulting in an increase in the profit of the EV manufacturer. In addition, the EV retailer’s profit also improves as demand increases. For the third-party collector, the EV manufacturer’s transfer price remains unchanged, while the average collection cost decreases; therefore, the profit increases. Overall, third-party economies of scale improve the profit of all members and the supply chain.

There are situations in which the profit of the third-party collector is negative. This means that in the M&TP mode, excessively high collection competition between the EV manufacturer and the third-party collector will cause the third-party collector to quit collection work. Therefore, the supply chain evolves into a single-channel collection mode.

The third-party collector prefers to quit collection work when the profit is less than 0. From the profit function of the third-party collector, it can be derived that the third-party collector will opt out of the collection market when $\delta > {1/{[\alpha (2 + \alpha )^{2} ]}}$.

When ${1/{[\alpha (2 + \alpha )^{2} ]}}{ = }1$, the critical point can be derived, that is, $\alpha^{*} { = }{1/3}( - 4 + ({{43}/2} - {{3\sqrt {177} } /2})^{{{1/3}}} + ({{43}/2}{ + }{{3\sqrt {177} } /2})^{{{1/3}}} )$. If $\alpha < \alpha^{*}$, then ${1/{[\alpha (2 + \alpha )^{2} ]}} > 1$, and because $\delta < 1$, we can conclude that if the competition is weak $\alpha < \alpha^{*}$, the third-party collector will not choose to exit the collection market, which means that in this situation the supply chain has dual collection channels.

**Figure 6:**
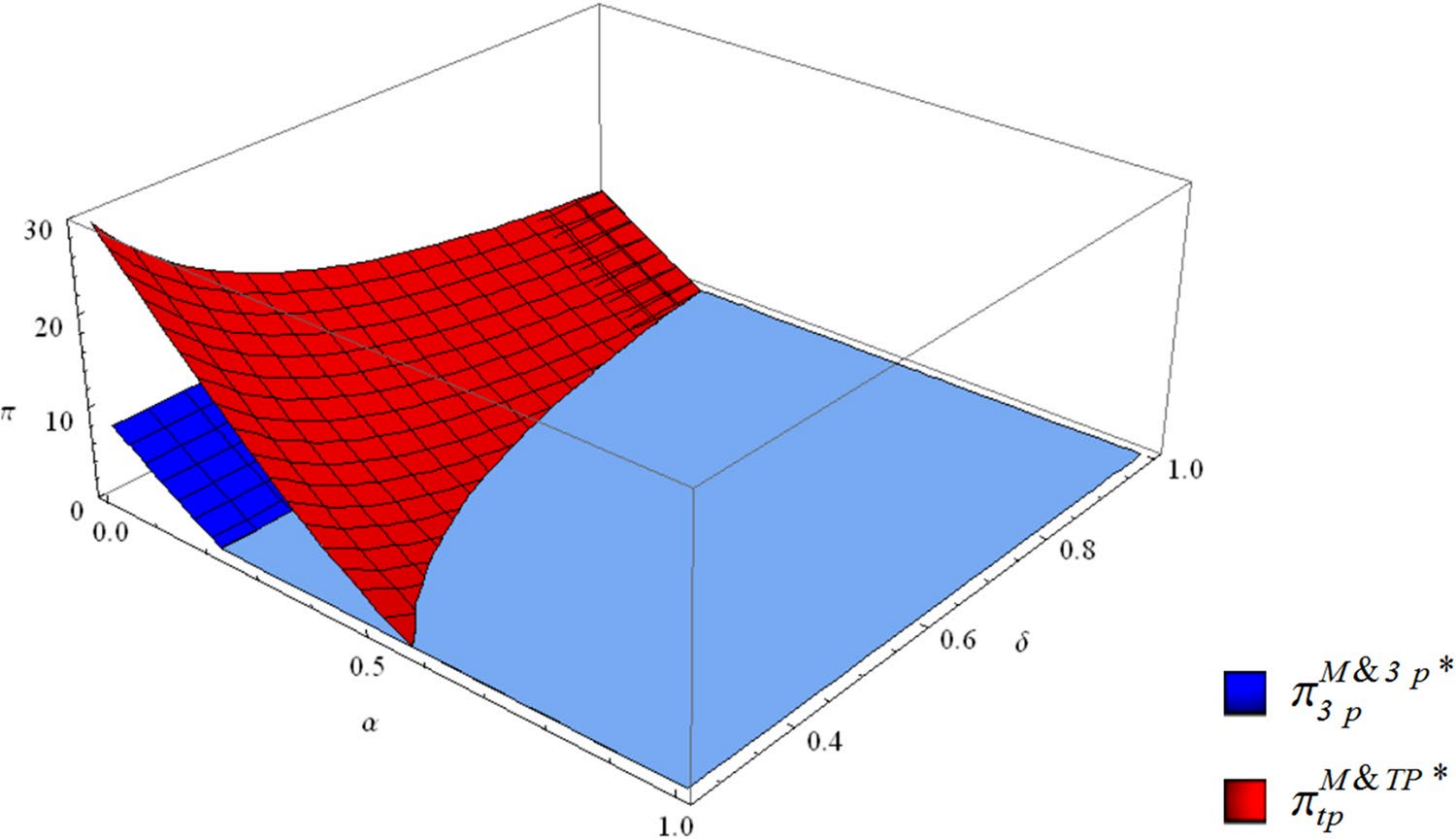
The third-party collector’s critical line with or without economies of scale.

With the increasing intense competition between the EV manufacturer and the third-party collector $\alpha^{*} < \alpha < 1$, only if the parameter is small enough $0 < \delta < {1/{[\alpha (2 + \alpha )^{2} ]}}$, will the third-party collector engage in collection activities. Otherwise, the third-party collector will quit collection. In this situation, the supply chain will evolve into a single-channel collection mode. The critical line of the third-party collector opting out of collection can be seen in Fig. 6.

Compared with the situation where third-party economies of scale are relatively low, stronger collection competition could force the third-party collector to withdraw from the collection market when the third-party economies of scale are higher. Third-party economies of scale can compensate for the increase in collection investment caused by collection competition. Hence, to some extent, economies of scale make the third-party collector more motivated to engage in collection activities.

#### Impact on the R&TP mode

##### The price decisions

###### **Corollary 7**

*The wholesale price, retail price, and transfer price in the two cases (with or without third-party economies of scale) are related as follows:* $w^{R\& 3P*} > w^{R\& TP*}$, $p^{R\& 3P*} > p^{R\& TP*}$, $b^{R\& 3P*} > b^{R\& TP*}$.

###### *Proof*

See supplementary materials, Proof of Corollary 7.

This shows the third-party economies of scale lead to a decrease in the retail price, wholesale price, and transfer price. Third-party economies of scale increase the total collection rate, resulting in a decline in the average production cost, which in turn causes the EV manufacturer and the EV retailer to lower the wholesale price and the retail price. However, in the R&TP mode, third-party economies of scale result in a decrease in the EV manufacturer’s transfer price.

##### The collection rate decisions

###### **Corollary 8**

*The collection rates of the retailer, the third-party collector, and the supply chain are related as follows:* $\tau_{r}^{R\& 3P*} > \tau_{r}^{R\& TP*}$, $\tau_{3p}^{R\& 3P*} < \tau_{tp}^{R\& TP*}$, and $\tau_{T}^{R\& 3P*} < \tau_{T}^{R\& TP*}$.

###### *Proof*

See supplementary materials, Proof of Corollary 8.

This indicates that third-party economies of scale decrease the collection rate of the EV retailer while increasing that of the third-party collector. Overall, the total collection rate is improved.

Third-party economies of scale decrease the average collection cost, which makes the third-party collector superior in the game with the retailer, resulting in the EV retailer lowering the collection rate. Furthermore, the marginal cost of increasing the collection rate for the third-party collector is much lower than that of the EV retailer. Therefore, the overall collection rate of the supply chain still increases, even though the collection rate of the EV retailer declines.

##### The profits of each member and the supply chain

**Figure 7:**
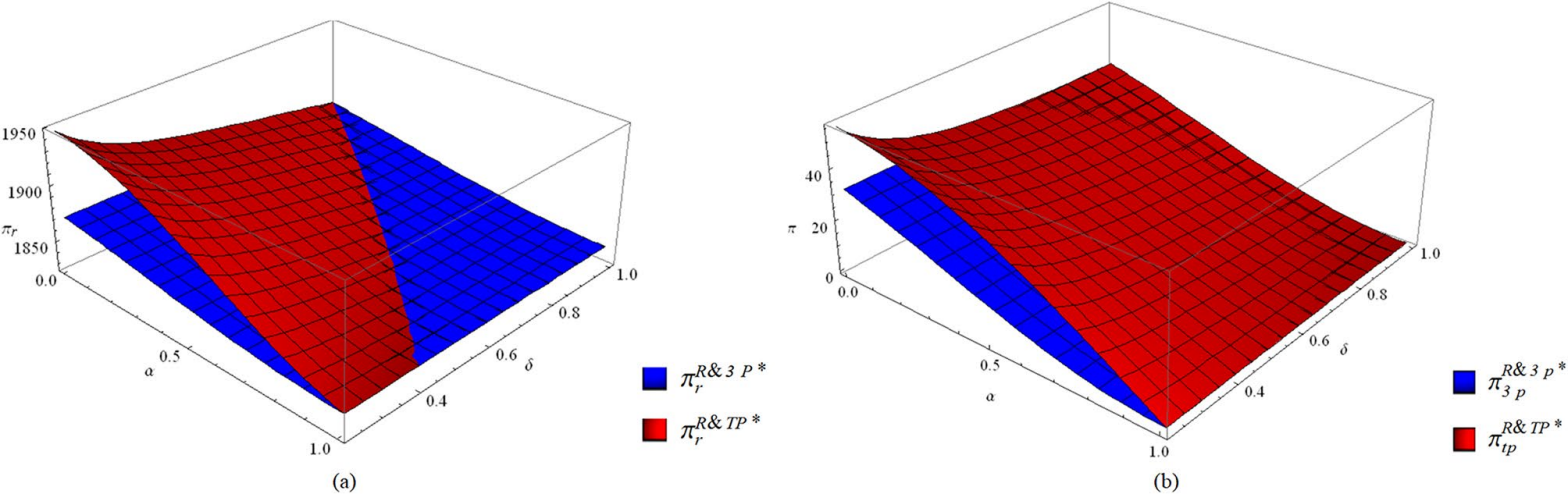
The profits of the retailer (**a**) and the third-party collector (**b**) with or without third-party economies of scale.

**Figure 8:**
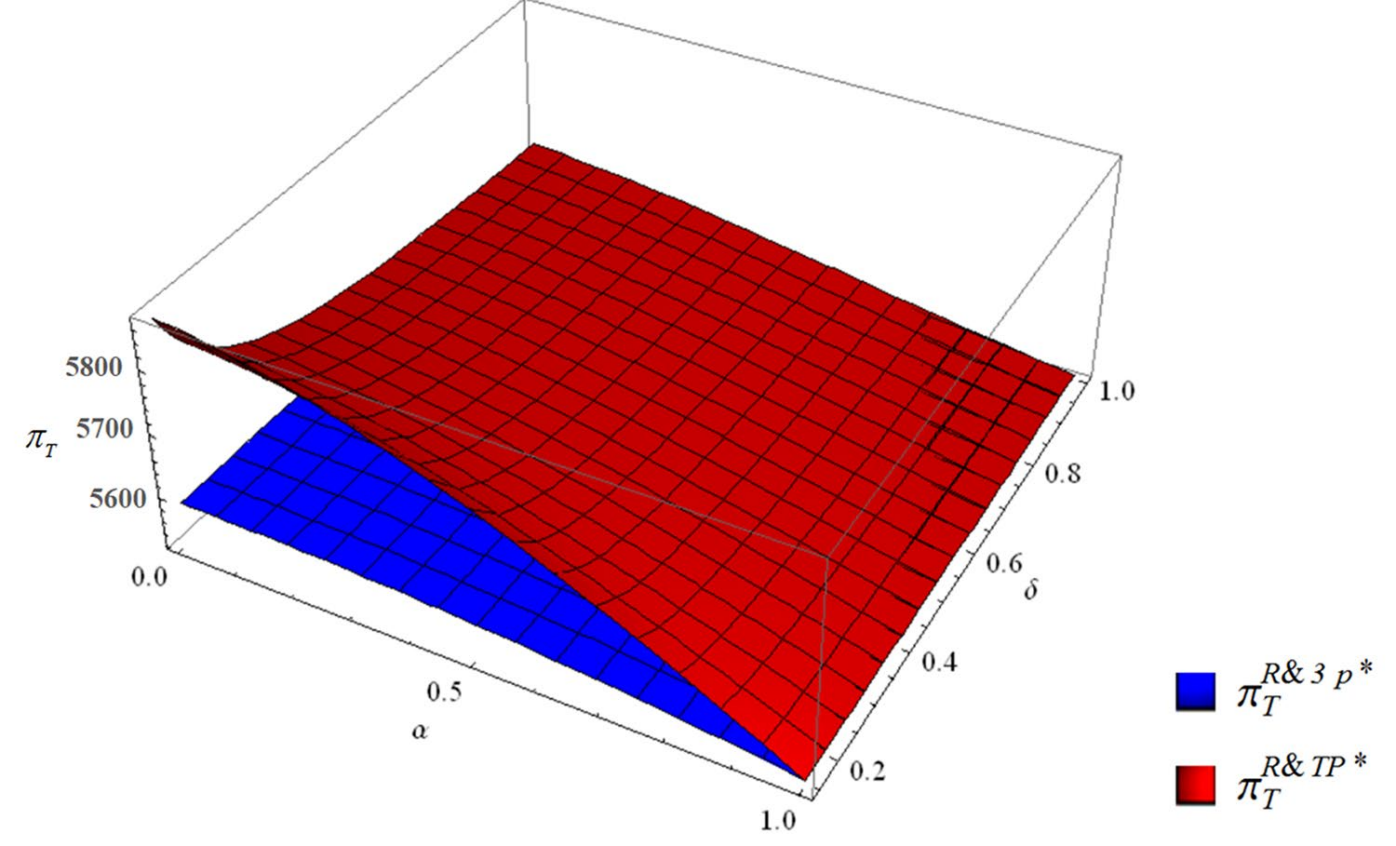
The total profits with or without third-party economies of scale.

Third-party economies of scale lead to an increase in the profit of the EV manufacturer (Eq. (28)). When collection competition becomes weak or third-party economies of scale are high, the profit of the retailer shows a rising trend (Fig. 7a). From the perspective of the third-party collector, the third-party economies of scale always improve the profit (Fig. 7b). For the supply chain, third-party economies of scale always increase the profit (Fig. 8).

28 $$\pi_{m}^{R\& 3P*} - \pi_{m}^{R\& TP*} { = } - \frac{{C(1 - \alpha^{2} )(1 - \delta )^{2} \Delta^{2} (f - \beta c_{m} )^{2} }}{{(4C - (1 - \alpha^{2} )\beta \Delta^{2} )(16C\delta - (1 - \alpha^{2} )\beta (1 + \delta )^{2} \Delta^{2} )}} < 0$$

The impact of third-party economies of scale on the EV manufacturer’s profit is consistent with that of the M&TP mode. For the EV retailer, although the collection investment decreases along with the decrease in the collection rate, the reduction in collection expense cannot compensate for the profit loss due to the falling price; therefore, the EV retailer’s profit is reduced (Fig. 7a).

From the perspective of the third-party collector, although third-party economies of scale reduce the unit transfer price paid by the EV manufacturer, they increase the collection rate of the third-party collector, and the increase in the total transfer payment is enough to compensate for the increased collection cost generated by the increased collection rate. In general, the profit of the third-party collector is improved (Fig. 7b).

From Fig. 8, for the whole supply chain, the total profit can be improved by an increase in the EV manufacturer’s and the third-party collector’s profits, although the profits of the EV retailer may not be improved in some scenarios.

## Conclusions

In this paper, three collection modes with dual competitive reverse channels were investigated. Competition and the different collection difficulties of the collectors are simultaneously introduced into the game models. The optimal pricing and collection effort decisions of different collection modes are obtained and compared. The collection mode choice strategy and the effects of third-party economies of scale are explored. Through the analysis, some interesting insights are derived as follows:

i. Third-party economies of scale can efficiently improve the collection rate and profit of the supply chain.
ii. No matter from the viewpoint of the collection rate or profit, the optimal collection mode depends on the competition intensity between different collection channels and third-party economies of scale.
iii. The M&R collection mode outperforms the M&TP and R&TP modes when the competition intensity is weak and third-party economies of scale are low. The R&TP mode will be the optimal collection mode only if third-party economies of scale are high enough.

The results in this paper can provide the EV battery supply chain with a certain reference to choose the optimal collection mode. However, there are still some limitations in this paper. Further research pursues two directions: (i) Information asymmetry among supply chain members should be considered. (ii) Attention should be devoted to the cooperation between different collection channels.

## Supplementary Information

Supplementary Information.

## Data Availability

All data generated or analyzed during this study are included in this published article.

## References

[1] Baars J, Domenech T, Bleischwitz R, Melin HE, Heidrich O (2021). Circular economy strategies for electric vehicle batteries reduce reliance on raw materials. Nat. Sustain..

[2] Fallah N, Fitzpatrick C, Killian S, Johnson M (2021). End-of-life electric vehicle battery stock estimation in Ireland through integrated energy and circular economy modelling. Resour. Conserv. Recyl..

[3] Kotak Y (2021). End of electric vehicle batteries: Reuse vs. recycle. Energies.

[4] Sakunai T, Ito L, Tokai A (2021). Environmental impact assessment on production and material supply stages of lithium-ion batteries with increasing demands for electric vehicles. J. Mater. Cycles Waste.

[5] IDTechEx. Second-Life Electric Vehicle Batteries 2020–2030. https://www.idtechex.com/en/research-report/second-life-electric-vehicle-batteries-2020-2030/681 (2019).

[6] Choi Y, Rhee S (2020). Current status and perspectives on recycling of end-of-life battery of electric vehicle in Korea (republic of). Waste Manag..

[7] Sun M, Yang X, Huisingh D, Wang R, Wang Y (2015). Consumer behavior and perspectives concerning spent household battery collection and recycling in China: A case study. J. Clean. Prod..

[8] Kim H, Jang Y, Hwang Y, Ko Y, Yun H (2018). End-of-life batteries management and material flow analysis in South Korea. Front. Environ. Sci. Eng..

[9] MIIT. The Interim Measures for the Administration of Recycling and Utilization of New Energy Vehicles Power Battery, https://www.miit.gov.cn/zwgk/zcwj/wjfb/zh/art/2020/art_459b0eb972964f68930bb39be9e92688.html (2018).

[10] Lyu X, Xu Y, Sun D (2021). An evolutionary game research on cooperation mode of the NEV power battery recycling and gradient utilization alliance in the context of China's NEV power battery retired tide. Sustainability.

[11] Zhang Q, Tang Y, Bunn D, Li H, Li Y (2021). Comparative evaluation and policy analysis for recycling retired EV batteries with different collection modes. Appl. Energy.

[12] Tang Y, Zhang Q, Li Y, Wang G, Li Y (2018). Recycling mechanisms and policy suggestions for spent electric vehicles' power battery—a case of Beijing. J. Clean. Prod..

[13] Ordoñez J, Gago EJ, Girard A (2016). Processes and technologies for the recycling and recovery of spent lithium-ion batteries. Renew. Sustain. Energy. Rev..

[14] Wang Y (2021). Recent progress on the recycling technology of Li-ion batteries. J. Energy Chem..

[15] Langner T, Sieber T, Acker J (2021). Studies on the deposition of copper in lithium-ion batteries during the deep discharge process. Sci. Rep..

[16] Ahmadi L, Young SB, Fowler M, Fraser RA, Achachlouei MA (2017). A cascaded life cycle: Reuse of electric vehicle lithium-ion battery packs in energy storage systems. Int. J. Life Cycle Ass..

[17] Wu W, Lin B, Xie C, Elliott RJR, Radcliffe J (2020). Does energy storage provide a profitable second life for electric vehicle batteries?. Energy Econ..

[18] Heymans C, Walker SB, Young SB, Fowler M (2014). Economic analysis of second use electric vehicle batteries for residential energy storage and load-levelling. Energy Policy.

[19] Sun B (2020). Economic analysis of lithium-ion batteries recycled from electric vehicles for secondary use in power load peak shaving in China. J. Clean. Prod..

[20] Sathre R, Scown CD, Kavvada O, Hendrickson TP (2015). Energy and climate effects of second-life use of electric vehicle batteries in California through 2050. J. Power Sources.

[21] Qiao D, Wang G, Gao T, Wen B, Dai T (2021). Potential impact of the end-of-life batteries recycling of electric vehicles on lithium demand in China: 2010–2050. Sci. Total Environ..

[22] Chen M (2019). Closed loop recycling of electric vehicle batteries to enable ultra-high quality cathode powder. Sci. Rep..

[23] Yu H (2021). Key technology and application analysis of quick coding for recovery of retired energy vehicle battery. Renew. Sustain. Energ. Rev..

[24] Tang Y (2019). The social-economic-environmental impacts of recycling retired EV batteries under reward-penalty mechanism. Appl. Energy.

[25] Han X, Wu H, Yang Q, Shang J (2016). Reverse channel selection under remanufacturing risks: Balancing profitability and robustness. Int. J. Prod. Econ..

[26] Yi P, Huang M, Guo L, Shi T (2016). Dual recycling channel decision in retailer oriented closed-loop supply chain for construction machinery remanufacturing. J. Clean. Prod..

[27] Lu Y, Xu X, Ai X (2018). Collective decision-making of a closed-loop supply chain dual-channel model under the third-party economies of scale. J. Ind. Eng. Eng. Manag..

[28] Savaskan RC, Bhattacharya S, Van Wassenhove LN (2004). Closed-loop supply chain models with product remanufacturing. Manag. Sci..

[29] Hong I, Yeh J (2012). Modeling closed-loop supply chains in the electronics industry: A retailer collection application. Transport. Res. E-Log..

[30] Wang W, Zhang Y, Li Y, Zhao X, Cheng M (2017). Closed-loop supply chains under reward-penalty mechanism: Retailer collection and asymmetric information. J. Clean. Prod..

[31] Hong X, Wang Z, Wang D, Zhang H (2013). Decision models of closed-loop supply chain with remanufacturing under hybrid dual-channel collection. Int. J. Adv. Manuf. Technol..

[32] Modak NM, Modak N, Panda S, Sana SS (2018). Analyzing structure of two-echelon closed-loop supply chain for pricing, quality and recycling management. J. Clean. Prod..

[33] Savaskan RC, Van Wassenhove LN (2006). Reverse channel design: The case of competing retailers. Manag. Sci..

[34] De Giovanni P (2018). A joint maximization incentive in closed-loop supply chains with competing retailers: The case of spent-battery recycling. Eur. J. Oper. Res..

[35] Xu C, Li B, Lan Y, Tang Y (2014). A closed-loop supply chain problem with retailing and recycling competition. Abstr. Appl. Anal..

[36] Huang M, Song M, Lee LH, Ching WK (2013). Analysis for strategy of closed-loop supply chain with dual recycling channel. Int. J. Prod. Econ..

[37] Wang W, Zhou S, Zhang M, Sun H, He L (2018). A closed-loop supply chain with competitive dual collection channel under asymmetric information and reward–penalty mechanism. Sustainability.

[38] Zhao J, Wei J, Li M (2017). Collecting channel choice and optimal decisions on pricing and collecting in a remanufacturing supply chain. J. Clean. Prod..

[39] Liu L, Wang Z, Xu L, Hong X, Govindan K (2017). Collection effort and reverse channel choices in a closed-loop supply chain. J. Clean. Prod..

[40] Liu Y, Zhang Y (2018). Closed loop supply chain under power configurations and dual competitions. Sustainability.

[41] Atasu A, Toktay LB, Van Wassenhove LN (2013). How collection cost structure drives a manufacturer's reverse channel choice. Prod. Oper. Manag..

[42] Chuang C, Wang CX, Zhao Y (2014). Closed-loop supply chain models for a high-tech product under alternative reverse channel and collection cost structures. Int. J. Prod. Econ..

[43] Huang M, Yi P, Guo L, Shi T (2015). Multi-dimensional reverse channel decision under different collection strategies. IFAC-PapersOnLine.

[44] Toyasaki F, Boyacι T, Verter V (2011). An analysis of monopolistic and competitive take-back schemes for WEEE recycling. Prod. Oper. Manag..

[45] Huang M, Yi P, Shi T (2017). Triple recycling channel strategies for remanufacturing of construction machinery in a retailer-dominated closed-loop supply chain. Sustainability.

