# Supplementary material for: Collection mode choice of spent electric vehicle batteries: considering collection competition and third-party economies of scale
Source: Sci Rep. 2022 Apr 23;12:6691. doi: 10.1038/s41598-022-10433-3 (PMC9035162; doi:10.1038/s41598-022-10433-3)
Supplement: Supplementary file 1 — Supplementary Information. [file 41598_2022_10433_MOESM1_ESM.pdf]

Collection mode choice of spent electric vehicle batteries: considering collection competition and third-party economies of scale

Xin Li [1]*

[1] School of Management Engineering, Shandong Jianzhu University, Jinan 250101, China

*** Corresponding author:**

Xin Li (lixin20@sdjzu.edu.cn)

Proof of Proposition 1

From Eqs. (3)-(4), we derive $\frac{\partial^2 \pi_r^{M\&TP}}{\partial p^2} = -2\beta < 0$ and $\frac{\partial^2 \pi_{tp}^{M\&TP}}{\partial \tau_{tp}^2} = -\frac{2C\delta}{1-\alpha^2} < 0$, hence, $\pi_r^{M\&TP}$ is concave in p and $\pi_{tp}^{M\&TP}$ is concave in τ_{tp}.

Using the first-order conditions of Eqs. (3)-(4), we have

$$\begin{cases} p^{M\&TP} = \dfrac{\phi + \beta w}{2\beta} \\ \tau_{tp}^{M\&TP} = \dfrac{b(1-\alpha^2)(\phi - \beta w)}{4C\delta} \end{cases} \tag{S1}$$

Substituting Eq. (S1) into Eq. (2), for given $b^{M\&TP}$, we have the Hessian matrix of $\pi_m^{M\&TP}$ with respect to w and τ_m.

$$H = \begin{bmatrix} \dfrac{\partial^2 \pi_m^{M\&TP}}{\partial w^2} & \dfrac{\partial^2 \pi_m^{M\&TP}}{\partial w \partial \tau_m} \\ \dfrac{\partial^2 \pi_m^{M\&TP}}{\partial \tau_m \partial w} & \dfrac{\partial^2 \pi_m^{M\&TP}}{\partial {\tau_m}^2} \end{bmatrix} = \begin{bmatrix} -\beta - \dfrac{\beta(b(1-\alpha^2)\beta(b(2+\alpha)-2\Delta))}{8C\delta} & -\dfrac{\beta\Delta}{2}) \\ -\dfrac{\beta\Delta}{2} & -\dfrac{2C}{1-\alpha^2} \end{bmatrix}$$

Since $0 < \alpha < 1$, When $b(2+\alpha) - 2\Delta > 0$, then the first order principal minor of H is less than zero, $-\beta - \frac{b\beta^2(1-\alpha^2)[b(2+\alpha)-2\Delta]}{8C} < 0$. When $8C\delta - \beta(1-\alpha^2)(\delta\Delta^2 + 2b\Delta - 2b^2 - \alpha b^2) > 0$, the second order principal minor of Hessian $\frac{\beta[8C\delta - \beta(1-\alpha^2)(\delta\Delta^2 + 2b\Delta - 2b^2 - \alpha b^2)]}{4(1-\alpha^2)\delta} > 0$. Hence, $\pi_m^{M\&TP}$ is jointly concave in w and τ_m.

From the first-order conditions of $\pi_m^{M\&TP}$ with respect to w and τ_m, we have

$$\begin{cases} w^{M\&TP} = \dfrac{4C\delta(\phi + \beta c_m) - \beta\phi(1-\alpha^2)(\delta\Delta^2 + 2\Delta b - 2b^2 - \alpha b^2)}{\beta[8C\delta - \beta(1-\alpha^2)(\delta\Delta^2 + 2\Delta b - 2b^2 - \alpha b^2)]} \\ \tau_m^{M\&TP} = \dfrac{\delta\Delta(1-\alpha^2)(\phi - \beta c_m)}{8C\delta - \beta(1-\alpha^2)(\delta\Delta^2 + 2\Delta b - 2b^2 - \alpha b^2)} \end{cases} \tag{S2}$$

Substituting Eq. (S2) into Eq. (S1), we get

$$\begin{cases} p^{M\&TP} = \dfrac{2C\delta(3\phi + \beta c_m) - \beta\phi(1-\alpha^2)(\Delta^2 + 2\Delta b - 2b^2 - \alpha b^2)}{\beta[8C\delta - \beta(1-\alpha^2)(\delta\Delta^2 + 2\Delta b - 2b^2 - \alpha b^2)]} \\ \tau_{tp}^{M\&TP} = \dfrac{b(1-\alpha^2)(\phi - \beta c_m)}{8C\delta - \beta(1-\alpha^2)(\delta\Delta^2 + 2\Delta b - 2b^2 - \alpha b^2)} \end{cases} \tag{S3}$$

Substituting Eq. (S2) and Eq. (S3) into Eq. (4) $\pi_m^{M\&TP}$, we can obtain:

$$\pi_m^{M\&TP} = \frac{C\delta(\phi-\beta c_m)^2}{\beta[8C\delta-\beta(1-\alpha^2)(\delta\Delta^2+2\Delta b-2b^2-\alpha b^2)]} \tag{S4}$$

From $\frac{\partial \pi_m^{M\&TP}}{\partial b^{M\&TP}} = \frac{-2C\delta(b(2+\alpha)-\Delta)(\phi-\beta c_m)^2(1-\alpha^2)}{(8C\delta-\beta(1-\alpha^2)(\delta\Delta^2+2b\Delta-b^2(2+\alpha)))^2} < 0$, we can easily prove that:

$$\begin{cases} \frac{\partial \pi_m^{M\&TP}}{\partial b^{M\&TP}} > 0 \quad if\ b^{M\&TP} \in (0, \frac{\Delta}{2+\alpha}) \\ \frac{\partial \pi_m^{M\&TP}}{\partial b^{M\&TP}} < 0 \quad if\ b^{M\&TP} \in (\frac{\Delta}{2+\alpha}, \Delta) \\ \frac{\partial \pi_m^{M\&TP}}{\partial b^{M\&TP}} = 0 \quad if\ b^{M\&TP} = \frac{\Delta}{2+\alpha} \end{cases}$$

Hence, the manufacturer can achieve the maximum profit when $b^{M\&TP*} = \Delta/(2+\alpha)$.

Substituting $b^{M\&TP*} = \Delta/(2+\alpha)$ into Eq. (S2) and Eq. (S3), we have the optimal decisions of the M&TP mode in Eqs. (5)-(9) and the total collection rate and maximum profits in Eqs. (10)-(14).

According to Eq. (11) and Eq. (13), we can easily verify that

$$\frac{\partial \pi_m^{M\&TP}}{\partial \tau_m^{M\&TP}}\Big|_{\tau_m^{M\&TP}=1-\tau_{tp}^{M\&TP}} = \frac{\partial \pi_m^{M\&TP}}{\partial \tau_m^{M\&TP}}\Big|_{\tau_{tp}^{M\&TP}=1-\tau_m^{M\&TP}}$$

$$= -\frac{2C\left(8C(2+\alpha)\delta-(1-\alpha^2)(1+(2+\alpha)\delta)\Delta(\beta\Delta+\phi)+(1-\alpha^2)\beta(1+(2+\alpha)\delta)\Delta c_m\right)}{(1-\alpha^2)\left(8C(2+\alpha)\delta-(1-\alpha^2)\beta(1+(2+\alpha)\delta)\Delta^2\right)} < 0$$

Since $\frac{\partial^2 \pi_m^{M\&TP}}{\partial \tau_m^{M\&TP2}} = -\frac{2C}{1-\alpha^2} < 0$, $\frac{\partial^2 \pi_{tp}^{M\&TP}}{\partial \tau_{tp}^{M\&TP2}} = -\frac{2C\delta}{1-\alpha^2} < 0$, we have $\frac{\partial \pi_m^{M\&TP}}{\partial \tau_m^{M\&TP}}\Big|_{\tau_m^{M\&TP}=1-\tau_{tp}^{M\&TP}} < \frac{\partial \pi_m^{M\&TP}}{\partial \tau_m^{M\&TP}}\Big|_{\tau_m^{M\&TP}=\tau_m^{M\&TP}} = 0$, and $\frac{\partial \pi_{tp}^{M\&TP}}{\partial \tau_{tp}^{M\&TP}}\Big|_{\tau_{tp}^{M\&TP}=1-\tau_m^{M\&TP}} < \frac{\partial \pi_{tp}^{M\&TP}}{\partial \tau_{tp}^{M\&TP}}\Big|_{\tau_{tp}^{M\&TP}=\tau_{tp}^{M\&TP}} = 0$. Therefore, the physical constraint $0 \le \tau_m^{M\&TP*} + \tau_{tp}^{M\&TP*} \le 1$ holds in the M&TP mode.

Proof of Proposition 2

From Eq. (16), we have the Hessian matrix of $\pi_r^{R\&TP}$ with respect to p and τ_r is as follows

$$H = \begin{bmatrix} -2\beta & -b\beta \\ -b\beta & -\frac{2C}{1-\alpha^2} \end{bmatrix}.$$

Since $\frac{\partial^2 \pi_r^{R\&TP}}{\partial p^2} = -2\beta < 0$ and $\frac{4\beta C}{1-\alpha^2} - b^2\beta^2 = \frac{\beta[4C-\beta b^2(1-\alpha^2)]}{1-\alpha^2} > 0$. Hence, $\pi_r^{R\&TP}$ is jointly concave in p and τ_r.

From the first-order conditions of Eq. (16), we have

$$\begin{cases} p^{R\&TP} = \frac{2C(\phi+\beta w) - b^2\phi\beta(1-\alpha^2)}{\beta[4C-b^2\beta(1-\alpha^2)]} \\ \tau_r^{R\&TP} = \frac{b(1-\alpha^2)(\phi-\beta w)}{4C-b^2\beta(1-\alpha^2)} \end{cases} \tag{S5}$$

Eq. (17) is concave in τ_{tp} since $\frac{\partial^2 \pi_{tp}^{R\&TP}}{\partial \tau_{tp}^2} = -\frac{2C\delta}{1-\alpha^2} < 0$.

Using the first-order conditions of Eq. (17), we get

$$\tau_{tp}^{R\&TP} = \frac{b(1-\alpha^2)(\phi - \beta w)}{[4C - b^2\beta(1-\alpha^2)]\delta} \tag{S6}$$

Substituting Eq. (S5) and Eq. (S6) into Eq. (15), and taking the second-order partial derivatives of $\pi_m^{R\&TP}$ for a given $b^{R\&TP}$, we have $\frac{\partial^2 \pi_m^{R\&TP}}{\partial w^2} = \frac{-4\beta C[4\delta C - b\beta((1+\delta)\Delta - b)(1-\alpha^2)]}{[4C - b^2\beta(1-\alpha^2)]^2\delta}$.

When $4\delta C - b\beta((1+\delta)\Delta - b)(1-\alpha^2) > 0$, then $\frac{-4\beta C[4\delta C - b\beta((1+\delta)\Delta - b)(1-\alpha^2)]}{[4C - b^2\beta(1-\alpha^2)]^2\delta} < 0$. Hence, $\pi_m^{R\&TP}$ is concave in w.

Using the first-order conditions of $\pi_m^{R\&TP}$ with respect to w, we obtain

$$w^{R\&TP} = \frac{\phi}{\beta} - \frac{\delta(4C - b^2\beta(1-\alpha^2))(\phi - \beta c_m])}{2\beta(4C\delta - b\beta(1-\alpha^2)((1+\delta)\varDelta - b))} \tag{S7}$$

Substituting Eqs. (S5)-(S7) into Eq. (15) $\pi_m^{R\&TP}$ yields

$$\pi_m^{R\&TP} = \frac{C\delta(\phi - \beta c_m)^2}{2\beta[4C\delta - b\beta((1+\delta)\Delta - b)(1-\alpha^2)]} \tag{S8}$$

From $\frac{\partial \pi_m^{R\&TP}}{\partial b^{R\&TP}} = \frac{C\delta(\phi - \beta c_m)^2(1-\alpha^2)((1+\delta)\Delta - 2b)}{2[4C\delta - b\beta((1+\delta)\Delta - b)(1-\alpha^2)]^2}$, we can easily prove that

$$\begin{cases} \frac{\partial \pi_m^{R\&TP}}{\partial b^{R\&TP}} > 0 & if\ b^{R\&TP} \in (0, \frac{(1+\delta)\Delta}{2}) \\ \frac{\partial \pi_m^{R\&TP}}{\partial b^{R\&TP}} < 0 & if\ b^{R\&TP} \in (\frac{(1+\delta)\Delta}{2}, \Delta) \\ \frac{\partial \pi_m^{R\&TP}}{\partial b^{R\&TP}} = 0 & if\ b^{R\&TP} = \frac{(1+\delta)\Delta}{2} \end{cases}$$

Hence, the manufacturer can achieve the maximum profit when $b^{R\&TP*} = (1+\delta)\Delta/2$.

Substituting $b^{R\&TP*} = (1+\delta)\Delta/2$ into Eqs. (S5)-(S7), we have the optimal solutions of the R&TP mode in Eq. (18)-(22) and the total colleting rate and maximum profits in Eq. (23)-(27).

According to Eq. (25) and Eq. (26), we can easily verify that

$$\frac{\partial \pi_r^{R\&TP}}{\partial \tau_r^{R\&TP}}|_{\tau_r^{R\&TP}=1-\tau_{tp}^{R\&TP}} = -\frac{2C\left(16C\delta - \left(1-\alpha^2\right)\left(1+\delta\right)^2\Delta\left(\beta\Delta + \phi\right) + \left(1-\alpha^2\right)\beta\left(1+\delta\right)^2\Delta c_m\right)}{\left(1-\alpha^2\right)\left(16C\delta - \left(1-\alpha^2\right)\beta\left(1+\delta\right)^2\Delta^2\right)} < 0$$

$$\frac{\partial \pi_{tp}^{R\&TP}}{\partial \tau_{tp}^{R\&TP}}|_{\tau_{tp}^{R\&TP}=1-\tau_r^{R\&TP}} = -\frac{2C\delta\left(16C\delta - \left(1-\alpha^2\right)\left(1+\delta\right)^2\Delta\left(\beta\Delta + \phi\right) + \left(1-\alpha^2\right)\beta\left(1+\delta\right)^2\Delta c_m\right)}{\left(1-\alpha^2\right)\left(16C\delta - \left(1-\alpha^2\right)\beta\left(1+\delta\right)^2\Delta^2\right)} < 0$$

Since $\frac{\partial^2 \pi_r^{\mathrm{R\&TP}}}{\partial \tau_r^{\mathrm{R\&TP}2}} = -\frac{2C}{1-\alpha^2} < 0$, $\frac{\partial^2 \pi_{tp}^{\mathrm{R\&TP}}}{\partial \tau_{tp}^{\mathrm{R\&TP}2}} = -\frac{2C\delta}{1-\alpha^2} < 0$, we have $\frac{\partial \pi_r^{R\&TP}}{\partial \tau_r^{R\&TP}}|_{\tau_r^{R\&TP}=1-\tau_{tp}^{R\&TP}} < \frac{\partial \pi_r^{R\&TP}}{\partial \tau_r^{R\&TP}}|_{\tau_r^{R\&TP}=\tau_r^{R\&TP}} = 0$, and $\frac{\partial \pi_{tp}^{R\&TP}}{\partial \tau_{tp}^{R\&TP}}|_{\tau_{tp}^{R\&TP}=1-\tau_r^{R\&TP}} < \frac{\partial \pi_{tp}^{R\&TP}}{\partial \tau_{tp}^{R\&TP}}|_{\tau_{tp}^{R\&TP}=\tau_{tp}^{R\&TP}} = 0$. Therefore, the physical constraint $0 \le \tau_r^{R\&TP*} + \tau_{tp}^{R\&TP*} \le 1$ holds in the R&TP model.

Proof of Corollary 1

We have $p^{M\&TP*} - p^{R\&TP*} = \frac{2C(1-\alpha^2)\delta(\alpha + (2+\alpha)\delta^2)\Delta^2(\phi - \beta c_m)}{(16C\delta - (1-\alpha^2)\beta(1+\delta)^2\Delta^2)L}$, it is obvious that $p^{M\&TP*} - p^{R\&TP*} > 0$, namely $p^{M\&TP*} > p^{R\&TP*}$.

In addition, we have $p^{M\&TP*} - p^{M\&R*} = \frac{2C(1-\alpha^2)((4-\alpha^2)\delta-1)\Delta^2(\phi-\beta c_m)}{(8C-(3-\alpha)(1-\alpha^2)\beta\Delta^2)L}$, the value of $p^{M\&TP*} - p^{M\&R*}$ depends on $(4-\alpha^2)\delta-1$:

(i) If $(4-\alpha^2)\delta-1<0$ ($\delta<\frac{1}{4-\alpha^2}$), then $p^{M\&TP*} < p^{M\&R*}$;

(ii) If $(4-\alpha^2)\delta-1>0$ ($\delta>\frac{1}{4-\alpha^2}$), then $p^{M\&TP*} > p^{M\&R*}$.

Besides, we obtain $p^{M\&R*} - p^{R\&TP*} = \frac{2C(1-\alpha^2)(1-\delta(4-2\alpha-\delta))\Delta^2(\phi-\beta c_m)}{(8C-(3-\alpha)(1-\alpha^2)\beta\Delta^2)(16C\delta-(1-\alpha^2)\beta(1+\delta)^2\Delta^2)}$, and the value of $1-\delta(4-2\alpha-\delta)$ decides the value of $p^{M\&R*} - p^{R\&TP*}$:

(i) If $1-\delta(4-2\alpha-\delta)>0$ ($\delta<2-\alpha-\sqrt{3-4\alpha+\alpha^2}$), then $p^{M\&R*} > p^{R\&TP*}$;

(ii) If $1-\delta(4-2\alpha-\delta)<0$ ($\delta>2-\alpha-\sqrt{3-4\alpha+\alpha^2}$), then $p^{M\&R*} < p^{R\&TP*}$.

Combing above comparisons, we have the Corollary 1.

Proof of Corollary 2

The following equation can be easily obtained $\pi_m^{M\&TP*} - \pi_m^{R\&TP*} = \frac{-C(1-\alpha^2)\delta(\alpha+(2+\alpha)\delta^2)\Delta^2(\phi-\beta c_m)^2}{(16C\delta-(1-\alpha^2)\beta(1+\delta)^2\Delta^2)L} < 0$, this means $\pi_m^{M\&TP*} < \pi_m^{R\&TP*}$.

Since $\pi_m^{M\&R*} - \pi_m^{M\&TP*} = \frac{C(1-\alpha^2)((4-\alpha^2)\delta-1)\Delta^2(\phi-\beta c_m)^2}{(8C-(3-\alpha)(1-\alpha^2)\beta\Delta^2)L}$, the value of $\pi_m^{M\&R*} - \pi_m^{M\&TP*}$ depends on $(4-\alpha^2)\delta-1$, therefore, we have the following conclusion:

(i) If $(4-\alpha^2)\delta-1>0$ ($\delta>\frac{1}{4-\alpha^2}$), then $\pi_m^{M\&R*} > \pi_m^{M\&TP*}$;

(ii) If $(4-\alpha^2)\delta-1<0$ ($\delta>\frac{1}{4-\alpha^2}$), then $\pi_m^{M\&R*} < \pi_m^{M\&TP*}$.

Since $\pi_m^{M\&R*} - \pi_m^{R\&TP*} = \frac{C(1-\alpha^2)(\delta(4-2\alpha-\delta)-1)\Delta^2(\phi-\beta c_m)^2}{(8C-(3-\alpha)(1-\alpha^2)\beta\Delta^2)(16C\delta-(1-\alpha^2)\beta(1+\delta)^2\Delta^2)}$, the value of $\pi_m^{M\&R*} - \pi_m^{R\&TP*}$ depends on $\delta(4-2\alpha-\delta)-1$, then we have:

(i) If $\delta(4-2\alpha-\delta)-1<0$ ($\delta<2-\alpha-\sqrt{(3-\alpha)(1-\alpha)}$), then $\pi_m^{M\&R*} < \pi_m^{R\&TP*}$;

(ii) If $\delta(4-2\alpha-\delta)-1>0$ ($\delta>2-\alpha-\sqrt{(3-\alpha)(1-\alpha)}$), then $\pi_m^{M\&R*} > \pi_m^{R\&TP*}$.

Combing above comparisons, we have the Corollary 2.

Proof of Corollary 3

Using the results in section 4, we have

$\tau_T^{R\&TP*} - \tau_T^{M\&TP*} = \frac{8C(1-\alpha^2)\delta(\alpha+(2+\alpha)\delta^2)\Delta(\phi-\beta c_m)}{(16C\delta-(1-\alpha^2)\beta(1+\delta)^2\Delta^2)L}$, it is easy to see $\tau_T^{R\&TP*} > \tau_T^{M\&TP*}$.

In addition, $\tau_T^{R\&TP*} - \tau_T^{M\&R*} = \frac{(1-\alpha^2)\Delta(8C(1-\delta)^2-(1-\alpha)^2(1+\alpha)\beta(1+\delta)^2\Delta^2)(\phi-\beta c_m)}{(8C-(3-\alpha)(1-\alpha^2)\beta\Delta^2)(16C\delta-(1-\alpha^2)\beta(1+\delta)^2\Delta^2)}$, We can see the value of $\tau_T^{R\&TP*} - \tau_T^{M\&R*}$ depends on the value of $8C(1-\delta)^2-(1-\alpha)^2(1+\alpha)\beta(1+\delta)^2\Delta^2$. In order to simplify the analysis, we refer the data in the existing research. It is derived that if $\delta>\frac{120-\alpha-\alpha^2-22\sqrt{1-\alpha-\alpha^2}}{118+\alpha+\alpha^2}$, then $\tau_T^{M\&R*} > \tau_T^{R\&TP*}$; if $\delta<\frac{120-\alpha-\alpha^2-22\sqrt{1-\alpha-\alpha^2}}{118+\alpha+\alpha^2}$, then $\tau_T^{M\&R*} > \tau_T^{R\&TP*}$.

Similarly, we compare $\tau_T^{M\&R*}$ and $\tau_T^{M\&TP*}$, we have if $\delta > \frac{118+\alpha+\alpha^2}{240+118\alpha-3\alpha^2}$, then $\tau_T^{M\&R*} > \tau_T^{M\&TP*}$, if $\delta < \frac{118+\alpha+\alpha^2}{240+118\alpha-3\alpha^2}$, then $\tau_T^{M\&R*} < \tau_T^{M\&TP*}$.

Combing above comparisons, we have the Corollary 3.

Proof of Corollary 4

The optimal decisions and maximum profits of the M&3P and the R&3P mode in existing research are as following Table S1. Comparing the results and ours, we analyze the impacts of the third-party economies of scale.

Table S1 Optimal decisions and maximum profits in the M&3P and R&3P mode regardless of third-party collector's economies of scale

	**M&3P**	**R&3P**
w	$\frac{4C(2+\alpha)(\phi+\beta c_m)-\beta\Delta^2\phi(3+\alpha)(1-\alpha^2)}{\beta[8C(2+\alpha)-\beta\Delta^2(3+\alpha)(1-\alpha^2)]}$	$\frac{\phi+\beta c_m}{2\beta}$
p	$\frac{2C(2+\alpha)(3\phi+\beta c_m)-\beta\Delta^2\phi(3+\alpha)(1-\alpha^2)}{\beta[8C(2+\alpha)-\beta\Delta^2(3+\alpha)(1-\alpha^2)]}$	$\frac{[3C-\Delta^2\beta(1-\alpha^2)]\phi+C\beta c_m}{\beta[4C-\Delta^2\beta(1-\alpha^2)]}$
b	$\frac{\Delta}{2+\alpha}$	Δ
τ_r	N/A	$\frac{\Delta(1-\alpha^2)(\phi-\beta c_m)}{2[4C-\Delta^2\beta(1-\alpha^2)]}$
τ_m	$\frac{\Delta(2+\alpha)(1-\alpha^2)(\phi-\beta c_m)}{8C(2+\alpha)-\beta\Delta^2(3+\alpha)(1-\alpha^2)}$	N/A
τ_{tp}	$\frac{\Delta(1-\alpha^2)(\phi-\beta c_m)}{8C(2+\alpha)-\beta\Delta^2(3+\alpha)(1-\alpha^2)}$	$\frac{\Delta(1-\alpha^2)(\phi-\beta c_m)}{2[4C-\Delta^2\beta(1-\alpha^2)]}$
τ_T	$\frac{\Delta(3+\alpha)(1-\alpha^2)(\phi-\beta c_m)}{8C(2+\alpha)-\beta\Delta^2(3+\alpha)(1-\alpha^2)}$	$\frac{\Delta(1-\alpha^2)(\phi-\beta c_m)}{[4C-\Delta^2\beta(1-\alpha^2)]}$
π_m	$\frac{C(2+\alpha)(\phi-\beta c_m)^2}{\beta[8C(2+\alpha)-\beta\Delta^2(3+\alpha)(1-\alpha^2)]}$	$\frac{C(\phi-\beta c_m)^2}{2\beta[4C-\Delta^2\beta(1-\alpha^2)]}$
π_r	$\frac{4C^2(2+\alpha)^2(\phi-\beta c_m)^2}{\beta[8C(2+\alpha)-\beta\Delta^2(3+\alpha)(1-\alpha^2)]^2}$	$\frac{C(\phi-\beta c_m)^2[4C-\Delta^2\beta(1+\alpha)(1-\alpha^2)]}{4\beta[4C-\Delta^2\beta(1-\alpha^2)]^2}$
π_{tp}	$\frac{C\Delta^2[1-\alpha(2+\alpha)^2](\phi-\beta c_m)^2(1-\alpha^2)}{[8C(2+\alpha)-\beta\Delta^2(3+\alpha)(1-\alpha^2)]^2}$	$\frac{C(\phi-\beta c_m)^2\Delta^2(1-\alpha)(1-\alpha^2)}{4[4C-\Delta^2\beta(1-\alpha^2)]^2}$
π_T	$\frac{(12C^2(2+\alpha)^2-\beta\Delta^2C(1+\alpha)(\alpha^2+4\alpha+5)(1-\alpha^2))(\phi-\beta c_m)^2}{\beta[8C(2+\alpha)-\beta\Delta^2(3+\alpha)(1-\alpha^2)]^2}$	$\frac{C(\phi-\beta c_m)^2[3C-\Delta^2\beta(1-\alpha^2)]}{\beta[4C-\Delta^2\beta(1-\alpha^2)]^2}$

Since $0<\alpha<1$, $0<\delta<1$, we obviously have the following equations:

$$w^{M\&3P*}-w^{M\&TP*}=\frac{(1-\alpha^2)(2+\alpha)\Delta^2(4C(1-\delta))(\phi-\beta c_m)}{KL}>0,$$

$$p^{M\&3P*}-p^{M\&TP*}=\frac{2C(1-\alpha^2)(2+\alpha)\Delta^2(1-\delta)(\phi-\beta c_m)}{KL}>0,$$

$$b^{M\&3P*}=b^{M\&TP*}=\frac{\Delta}{2+\alpha}.$$

The Corollary 4 is proved.

Proof of Corollary 5

Comparing the results in this paper and the exist reference (see Table S1), then we have $\tau_m^{M\&3P*}-\tau_m^{M\&TP*}=\frac{(1-\alpha^2)^2(2+\alpha)\beta\Delta^3(1-\delta)(\phi-\beta c_m)}{KL}$. Since $0<\alpha<1$, $0<\delta<1$, therefore, $\tau_m^{M\&3P*}>\tau_m^{M\&TP*}$.

We have $\tau_{tp}^{M\&3P*}-\tau_{tp}^{M\&TP*}=-\frac{(1-\alpha^2)(2+\alpha)\Delta(1-\delta)(8C-(1-\alpha^2)\beta\Delta^2)(\phi-\beta c_m)}{KL}$, since $0<\alpha<1$, $0<\delta<1$ and the C is very high, $8C>(1-\alpha^2)\beta\Delta^2$, then $\tau_{tp}^{M\&3P*}-\tau_{tp}^{M\&TP*}<0$, namely $\tau_{tp}^{M\&3P*}<\tau_{tp}^{M\&TP*}$.

Since $0<\alpha<1$, $0<\delta<1$, we can easily derive that $\tau_T^{M\&3P*}-\tau_T^{M\&TP*}=-\frac{8C(1-\alpha^2)(2+\alpha)\Delta(1-\delta)(\phi-\beta c_m)}{KL}<0$.

The Corollary 5 is proved.

Proof of Corollary 6

Since $0<\alpha<1$, $0<\delta<1$, we have

$$\pi_m^{M\&3P*}-\pi_m^{M\&TP*}=-\frac{C(1-\alpha^2)(2+\alpha)\Delta^2(1-\delta)(\phi-\beta c_m)^2}{KL}<0,$$

$$\pi_r^{M\&3P*}-\pi_r^{M\&TP*}=-\frac{4C^2(1-\alpha^2)(2+\alpha)^2\Delta^2(1-\delta)(\phi-\beta c_m)^2H}{KL}<0,$$

$$\pi_T^{M\&3P*}-\pi_T^{M\&TP*}=-\frac{C(1-\alpha^2)(2+\alpha)\Delta^2(1-\delta)(\phi-\beta c_m)^2(1+4C(2+\alpha)H)}{KL}<0.$$

And, $H=16C(2+\alpha)\delta-(1-\alpha^2)\beta(1+(5+2\alpha)\delta)\Delta^2>0$.

The Corollary 6 is proved.

Proof of Corollary 7

Since $0<\alpha<1$, $0<\delta<1$, it is easy to see:

$$w^{R\&3P*}-w^{R\&TP*}=\frac{(1-\alpha^2)(1-\delta)(1+\delta)^2\Delta^2(\phi-\beta c_m)}{2(16C\delta-(1-\alpha^2)\beta(1+\delta)^2\Delta^2)}>0,$$

$$p^{R\&3P*}-p^{R\&TP*}=\frac{C(1-\alpha^2)(1-\delta)^2\Delta^2(\phi-\beta c_m)}{(4C-(1-\alpha^2)\beta\Delta^2)(16C\delta-(1-\alpha^2)\beta(1+\delta)^2\Delta^2)}>0,$$

$$b^{R\&3P*}-b^{R\&TP*}=\frac{(1-\delta)\Delta}{2}>0.$$

The Corollary 7 is proved.

Proof of Corollary 8

Since $0<\alpha<1$, $0<\delta<1$, and $8C\delta>(1-\alpha^2)\beta(1+\delta)\Delta^2$, then we have the following results:

$$\tau_r^{R\&3P*}-\tau_r^{R\&TP*}=\frac{(1-\alpha^2)(1-\delta)\Delta(8C\delta-(1-\alpha^2)\beta(1+\delta)\Delta^2)(\phi-\beta c_m)}{2(4C-(1-\alpha^2)\beta\Delta^2)(16C\delta-(1-\alpha^2)\beta(1+\delta)^2\Delta^2)}>0,$$

$$\tau_{tp}^{R\&3P*}-\tau_{tp}^{R\&TP*}=-\frac{(1-\alpha^2)(1-\delta)\Delta(8C-(1-\alpha^2)\beta(1+\delta)\Delta^2)(\phi-\beta c_m)}{2(4C-(1-\alpha^2)\beta\Delta^2)(16C\delta-(1-\alpha^2)\beta(1+\delta)^2\Delta^2)}<0,$$

$$\tau_T^{R\&3P*}-\tau_T^{R\&TP*}=-\frac{4C(1-\alpha^2)(1-\delta)^2\Delta(\phi-\beta c_m)}{(4C-(1-\alpha^2)\beta\Delta^2)(16C\delta-(1-\alpha^2)\beta(1+\delta)^2\Delta^2)}<0.$$

The Corollary 8 is proved.
